# Cryo-EM structures of an insecticidal Bt toxin reveal its mechanism of action on the membrane

**DOI:** 10.1038/s41467-021-23146-4

**Published:** 2021-05-14

**Authors:** Matthew J. Byrne, Matthew G. Iadanza, Marcos Arribas Perez, Daniel P. Maskell, Rachel M. George, Emma L. Hesketh, Paul A. Beales, Marc D. Zack, Colin Berry, Rebecca F. Thompson

**Affiliations:** 1 Astbury Centre for Structural and Molecular Biology, School of Molecular and Cellular Biology, Faculty of Biological Sciences, University of Leeds, Leeds, UK; 2 Astbury Centre for Structural and Molecular Biology, School of Chemistry, Faculty of Engineering and Physical Sciences & Astbury Centre for Structural and Molecular Biology, University of Leeds, Leeds, UK; 3 Corteva Agriscience, Indianapolis, IN USA; 4 Cardiff School of Biosciences, Cardiff University, Cardiff, UK; 5 Present Address: Scientific Computing Department, Science and Technology Facilities Council, Research Complex at Harwell, Didcot, UK

**Keywords:** Proteins, Cryoelectron microscopy, Cryoelectron tomography

## Abstract

Insect pests are a major cause of crop losses worldwide, with an estimated economic cost of $470 billion annually. Biotechnological tools have been introduced to control such insects without the need for chemical pesticides; for instance, the development of transgenic plants harbouring genes encoding insecticidal proteins. The Vip3 (vegetative insecticidal protein 3) family proteins from *Bacillus thuringiensis* convey toxicity to species within the Lepidoptera, and have wide potential applications in commercial agriculture. Vip3 proteins are proposed to exert their insecticidal activity through pore formation, though to date there is no mechanistic description of how this occurs on the membrane. Here we present cryo-EM structures of a Vip3 family toxin in both inactive and activated forms in conjunction with structural and functional data on toxin–membrane interactions. Together these data demonstrate that activated Vip3Bc1 complex is able to insert into membranes in a highly efficient manner, indicating that receptor binding is the likely driver of Vip3 specificity.

## Abstract (web summary)

The Vip3 family proteins from *Bacillus thuringiensis* are thought to exert their insecticidal activity through pore formation. Here authors present cryo-EM structures of a Vip3 family toxin in both inactive and activated forms and show the activated Vip3Bc1 in its pore forming conformation on the membrane.

## Introduction

Pest insects are a major cause of crop losses^1^ worldwide, both by direct damage to plants and via the spread of plant diseases that reduce yields. These losses threaten global food security and the economic viability of crop farming. Biotechnology is a rich source of strategies for controlling insect pests that, in turn, circumvent the use of chemical pesticides that damage the environment. Furthermore, the potential increase in yields and decrease in pesticide use have the potential to transform the carbon footprint of agriculture^2^. One approach that has gained considerable interest is to create transgenic plants expressing pesticidal proteins from the bacterium *Bacillus thuringiensis* (Bt). These proteins are active against many families of insect pests in a species and life-cycle specific manner, but have no activity against non-target organisms, therefore imparting highly specific insecticidal activity.

The majority of the pesticidal proteins utilised in transgenic crops to date belong to the 3-domain family of Cry toxins (3D-Cry) from Bt, which have been deployed commercially since the early 1990s^3^. This approach was initially successful, but resistance has arisen amongst target insects. Subsequently, efforts have been made to combine multiple toxins in ‘pyramids’ in order to combat resistance^4^. If this combinatorial approach is to be fully realised we need to understand more about the structure, function and mechanism of other families of pesticidal proteins, so they might be properly deployed synergistically. Furthermore, a molecular level understanding of toxin structure might facilitate rational redesign of Bt-proteins for increased specificity and/or potency.

Vip3 toxins are produced by Bt strains during the vegetative growth phase, and act in a species specific manner against insects in the order Lepidoptera, which includes some of the most damaging insect pests^5^. Vip3Aa variants have already seen success in commercial transgenic corn and cotton varieties^6^.

The Vip3 family of proteins bear no appreciable sequence homology with other toxins for which the atomic structures have been determined (including 3D-Cry), and as such, interrogation of their mechanism of toxicity is of particular interest.

The mechanism by which Vip3 proteins kill insects has been broadly characterised but the precise details remain unclear^7^. The Vip3 proteins have molecular masses of ~90 kDa, and are secreted as soluble proteins that form tetramers in solution^8^. They can be proteolytically processed by trypsin or insect gut extracts to yield products of ~65 kDa and ~21 kDa that remain closely associated in solution^8,9^. This proteolytic processing has been shown to be required for Vip3 cytotoxicity^10,11^. In the midgut of susceptible larvae, the toxin binds to receptors on target cells that are not yet fully characterised^12,13^. At some stage following receptor engagement, the Vip3 proteins are proposed to form pores in insect cell membranes^10^. These findings suggest that toxicity is likely to result from pore formation leading to cell necrosis, however, other mechanisms of toxicity have been suggested including the initiation of apoptosis^14^.

A flurry of recent structural information has been released for Vip3 complexes, including an X-ray crystallography structure of non-native, unprocessed VIP3B2160^15^, and cryoEM structures of Vip3Aa16 in its unprocessed and activated forms (released while this manuscript was under review)^16^. These structures, along with those presented here, offer insight into the architecture and mechanism of Vip3 activation and toxicity. Here, we reconstitute Vip3Bc1 activity in vitro and demonstrate its propensity to perturb membranes. Furthermore, we present the structure of wild-type Vip3Bc1 variant in its pre-processed (autoinhibited) and processed (activated)(Vip3Bc1^act)^ states and visualise Vip3Bc1^act^ inserted into liposomes via cryo-electron tomography (cryoET) allowing us to observe the activated form of the toxin in the biologically relevant environment of the membrane.

## Results

### Structure of Vip3Bc1

**Fig. 1:**
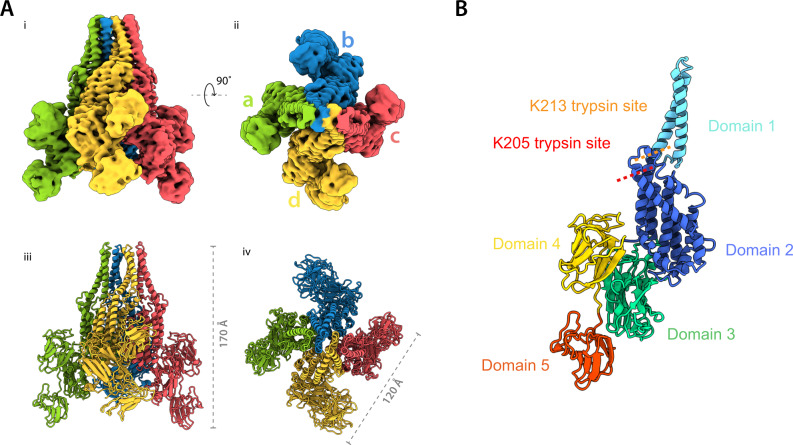
Structure of Vip3Bc1. **A** Quaternary structure of Vip3Bc1 tetramer as shown from two views of the complex related by 90° for the EM density (i, ii) and model (iii, iv). **B** Model of Vip3Bc1 illustrating domain structure as a monomer. The model shown corresponds to subunit A. Primary trypsin processing site K205 is indicated. Domain structure with respect to the primary sequence can be seen in Supplementary Fig. 4.

We first examined the structure of the wild type, full length Vip3Bc1 by single particle cryoEM (Fig. 1). Vip3Bc1 was recombinantly expressed in *Pseudomonas fluorescens* and purified as previously described^9^. It was determined to be monodisperse in solution by size exclusion chromatography (SEC), and a single peak was observed at the elution volume anticipated for a Vip3Bc1 tetramer (Supplementary Fig. 1). The 3D reconstruction of the tetrameric assembly of Vip3Bc1 with a global resolution of 3.9 Å resolution was obtained (0.143 FSC threshold) (Supplementary Figs. 2, 3). The architecture for the complex is consistent with that of the recently determined structures of mutant Vip3 variant Vip3B2160 and with the cryoEM structure of Vip3Aa16^15,16^.

Our Vip3Bc1 structure reveals a tetrameric assembly with C2 symmetry (Fig. 1). Each tetramer comprises two dimers formed by conformationally distinct A:B monomers. Each monomer of Vip3Bc1 consists of 5 domains (Fig. 1B). Together, domain 1 and domain 2 form the N-terminal, largely alpha-helical portion of the protein. We have defined domains based on the architecture of the unprocessed tetramer previously used^15^ (Supplementary Fig. 4). The two domains are connected by a contiguous alpha-helix (α3, residues 94-162) that interfaces with α2 (residues 61-88) undertaking a super-helical twist that gives the complex a distinct conical appearance. Domains 3-5 make up the C-terminal portion of the protein, primarily composed of β-strands. The interchain interactions that bring about the conical morphology and impart the C2 symmetry are formed at the interface between domains 1 and 2 on each of the monomers, with the rest of the complex not involved in oligomerisation. Using this cryoEM density map in conjunction with the recently deposited crystal structure coordinates of the mutant protein Vip3B2160 (PDB 6V1V), we generated a complete atomic model for each A:B monomer, which together with symmetry operations yield a model of the bioassembly.

### Proteolytic processing of Vip3Bc1

To investigate the structure and activity of proteolytically cleaved Vip3Bc1, we performed a trypsin digest to yield two primary digestion fragments of ~68 kDa and ~21 kDa respectively, which we term activated Vip3Bc1 (Vip3Bc1^act^) (Supplementary Fig. 1). Mass spectrometry analysis indicates that these fragments are formed primarily by digestion at K205 (Supplementary Fig. 5). This result differs to those observed in previous studies, which report Vip3Bc1 digestion with Lepidopteran midgut enzymes occurs primarily at K213^9^. This discrepancy could be attributed to the differing digestive enzymes used in each study, and could point to redundancy in the activation mechanism of Vip3Bc1, with cleavage at a number of locations on this loop yielding active toxin. Both of these cleavage sites are present on a solvent exposed loop of domain 2, allowing proteases to access these residues inside the insect gut. Consistent with previous reports, we saw no evidence for the first 20 N-terminal residues, which sit prior to the cleavage site (*) in the R*ALPSF site which is well conserved amongst all known subfamilies of Vip3 proteins (Supplementary Fig. 4)^9^.

**Fig. 2:**
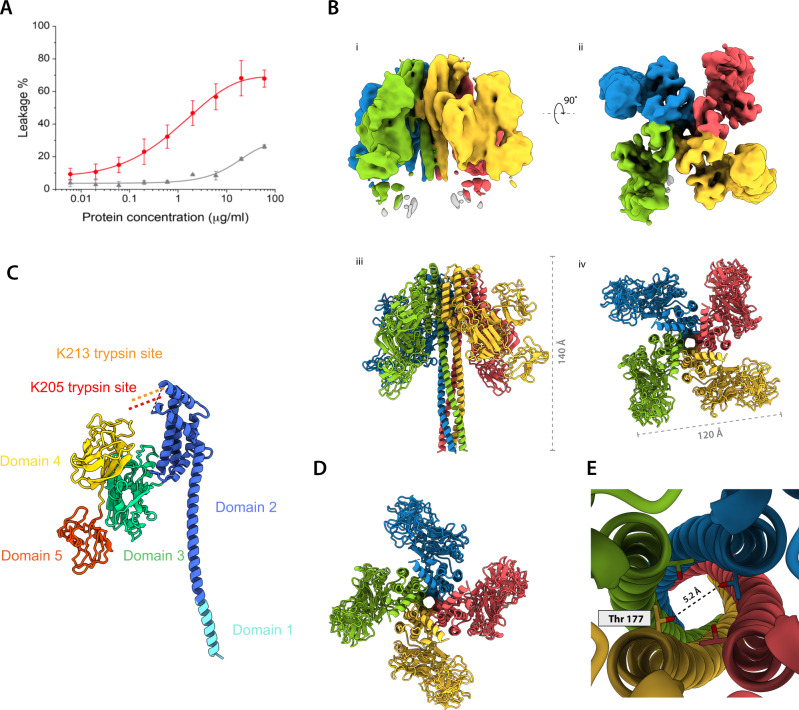
Vip3Bc1^act^ has a greater propensity to perturb liposome membranes compared with Vip3Bc1. **A** Digestion of the complex increases activity according to CF dye release from LUVs upon addition of Vip3Bc1^act^ (red) and Vip3Bc1 (grey). Plotted data represents the mean and the error bars the standard deviation of the measurements from three independent replicates (Full data shown Supplementary Table 3 and 4). **B** Quaternary structure of Vip3Bc1^act^ tetramer as shown from two views of the complex related by 90° for the EM density (I, ii) and model (iii, iv). **C** Model of Vip3Bc1^act^ asymmetric unit, illustrating domain structure as a monomer. **D** Vip3Bc1^act^ tetrameric assembly as shown from the top conformation is consistent with a pore. **E** Pore structure of Vip3Bc1^act^.

To investigate the differing propensity of Vip3Bc1 and Vip3Bc1^act^ to perturb membranes, dye release assays were performed, exposing DOPC and DOPE (6:4) unilamellar vesicles (LUVs) loaded with 5(6)-carboxyfluorescein (CF), to Vip3Bc1 and Vip3Bc1^act^ protein at varying concentrations. This lipid composition was chosen as insect membranes are highly enriched in PE^17^. Based on previous reports which state that Vip3Aa35 membrane perturbation in calcein release assays was of greatest magnitude at pH 8, and not active at pH 10 (ref. ^10^), we conducted our dye release assays at pH 8.5, and measured perturbation after 30 min in order to obtain ‘end point’ data. These data demonstrate that the digested Vip3Bc1^act^ has a much higher propensity to perturb LUVs than undigested protein (Fig. 2A), with >100-fold more Vip3Bc1 being required to achieve a similar magnitude of perturbation achieved by Vip3Bc1^act^. The activated toxin Vip3Bc1^act^ perturbs membranes in a dose dependent manner (Fig. 2A).

To interrogate the structural basis for VipBc1^act^ enhanced ability to perturb membranes, we analysed the complex by single particle cryoEM. We obtained a 3D reconstruction of the digested complex by single particle cryo-EM analysis to a global resolution of 4.8 Å (0.143 FSC threshold) (Fig. 2 B-C, Supplementary Figs. 3, 6). The structures reveal that Vip3Bc1 to VipBc1^act^ transition requires extensive conformational rearrangements, but consistent with previous biochemical analyses, retains its tetrameric state^8^ while opening an approximately 5 Å pore (Fig. 2 D,E). This structure is in marked contrast to the recently reported crystal structure of Vip3Aa11_200-end,_ (completely deleting the cleaved N-terminal region) where the truncation likely resulted in a non-native structure^18^. The Vip3Bc1^act^ dataset shows a strong preferred orientation compared with Vip3Bc1 (Supplementary Fig. 3); to generate our 3D structure we used a tilted collection strategy^19^. The shift in angular orientation distribution may be indicative of changes in surface charge/hydrophobicity altering the particles’ interactions with the air-water interface^20^. The preferred orientation produced a Vip3Bc1^act^ cryoEM map with pronounced anisotropy and of poorer quality than the recently reported cryoEM structure of activated Vip3Aa^16^. We were not able to directly observe density for an extended alpha-helical bundle in our single particle cryoEM reconstruction of Vip3Bc1^act^, however we were able to use deposited Vip3Aa16 coordinates to generate a model for Vip3Bc1^act^ EM density due to high sequence identity (59.9%).

**Fig. 3:**
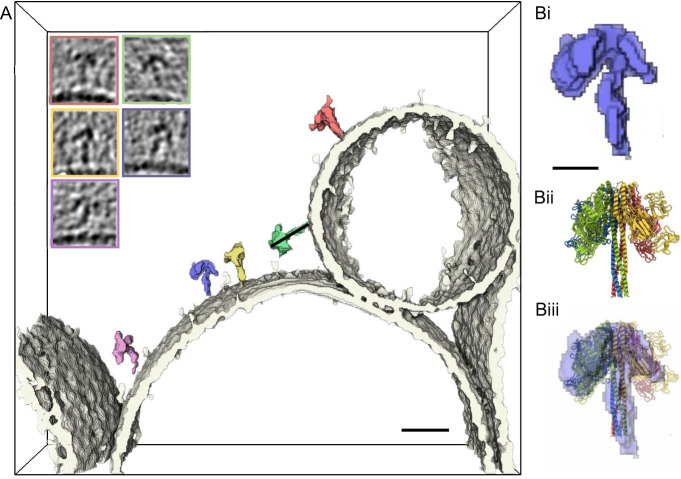
Vip3^act^ directly visualised on liposome membranes. Segmented density from cryo-ET shows interaction of Vip3Bc1^act^ (coloured) with the LUV membrane (white). Images inset show section through the tomogram of the matching particle in segmentation. Full tomogram can be visualised in Supplementary Movie 1. Scale bar 20 nm. **B** Scaled comparison between segmented density for a single particle in the tomogram, with 5 nm scale bar (i) compared to Vip3Bc1^act^ model (ii) with an overlay showing the stalk density fits the 4-helix bundle well, and the density which is distal to the membrane is consistent with single particle Vip3Bc1^act^ model. The helical stalk modelled in Vip3Bc1^act^ does not account for all of the stalk density observed in the sub-volume.

It has been widely proposed that Vip3 proteins impart their toxicity via pore formation. To inform our understanding of pore formation by Vip3Bc1, we used cryo-ET to directly observe Vip3Bc1^act^ interaction with the membrane. We prepared cryoEM grids of LUVs incubated with either Vip3Bc1 or Vip3Bc1^act^ and imaged using cryoET. We observed that Vip3Bc1^act^ associated with the membrane in an elongated ‘open umbrella’ conformation, ~15–18 nm on its longest dimension to the membrane, with a stalk interacting with the membrane and the bulk of the protein density distal to the membrane (Fig. 3, Supplementary Movie 1). These data support the model that activated Vip3 toxins interact with the membrane via an extended helical bundle, and is consistent with negative stain EM images of a Vip3Aa35^10^ and the recently published structure of Vip3Aa16^16^, suggesting that this conformational change upon digestion may be common across Vip3 variants. Inspection of the tomograms reveals the stalk penetrating the membrane (Supplementary Movie 2).

In addition to single Vip3Bc1^act^ tetramers imaged on the membrane, we also observed instances where individual liposomes were highly enriched in Vip3Bc1 particles (Supplementary Movie 3). However, in the majority of tilt series collected, we were unable to observe protein interacting with the membrane. In tomograms of LUVs exposed to undigested Vip3Bc1, we were not able to identify any Vip3Bc1 binding to the membrane, although protein aggregates were observed in some tomograms (Supplementary Movie 4).

## Discussion

### Vip3Bc1 domain structure

**Fig. 4:**
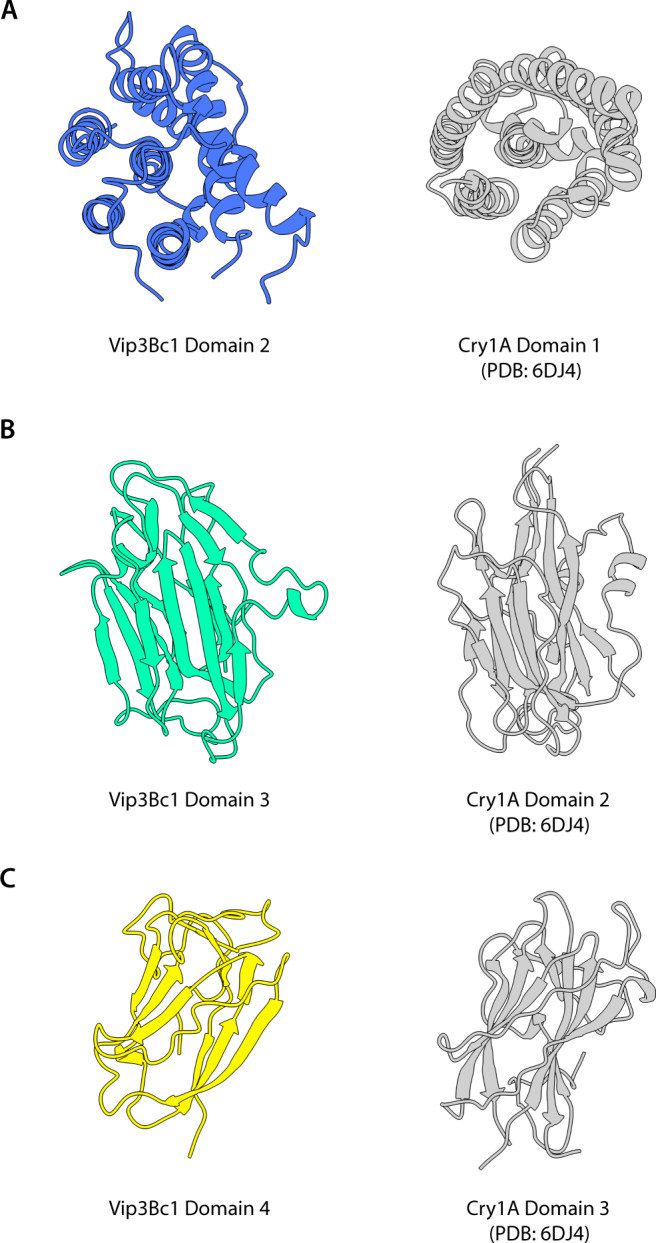
Structural homology of Vip3 domains to counterpart 3D-cry domain. Structural homology of Vip3 domains to counterpart 3D-cry domain. **A** Vip3Bc1 domain 2 and 3D-Cry domain 1 (Cry1A domain 1, PDB 6DJ4 shown) share a predominantly hydrophobic alpha helix surrounded by other helices in a bundle. **B** Vip3Bc1 domain 3 and 3D-Cry domain 2 (Cry1A domain 2 PDB 6DJ4) share a beta prism fold with three sides made up of antiparallel beta sheets. **C** Domains 4 Vip3Bc1 and 3D-Cry domain 3 (Cry1A domain 3, PDB 6DJ4 shown) share a twisted beta-sheet ‘jelly roll’ topology.

The liposome dye release data presented here support the hypothesis that activated Vip3 imparts its toxicity via membrane pore formation. In this respect, Vip3 is reminiscent of 3D-Cry proteins, which induce cell death through insertion into insect gut membranes, pore formation and ultimately cell death by lytic osmosis^21^. Whilst analysing our Vip3Bc1 domain structures, we observed that they bear striking structural homology to domains found in the 3D-Cry toxin family of proteins, despite sharing no similarity at the primary sequence level (Fig. 4). It is, therefore, reasonable to speculate that each of the domains in Vip3Bc1 may carry out similar activities to their counterpart component in the more widely studied 3D-Cry proteins.

Domain 2 of Vip3Bc1 shares the same protein fold as domain 1 of 3D-Cry proteins, which is proposed to play a role in membrane perturbation (Fig. 4). Each has a predominantly hydrophobic alpha helix surrounded by five other helices in a helical bundle. A proposed mode of action of 3D-Cry proteins involves the hinge movement of two helices of the helical bundle into a roughly equivalent position of α2 and α3, which form the conical tip of Vip3Bc1^22^. This suggests that this architecture is an integral part of the mode of action of both Vip3Bc1 and 3D-Cry toxins.

Vip3Bc1 domain 3 is structurally similar to domain 2 from 3D-Cry proteins, which has a role in receptor binding in 3D-Cry^23^. Both share a beta prism fold with three sides made up of antiparallel beta sheets (Fig. 4). Recent studies support the role of domain 3 in receptor recognition in Vip3^18^. The regions equivalent to the Vip3Bc1 domain 3 loop I488-T492 show high levels of variability between Vip3A, Vip3B and Vip3C proteins (Supplementary Fig. 4) but the significance of such loops to the target specificity of toxins remains to be established. Preceding this loop is a region where Vip3B toxins are distinct from other Vip3 variants in that they have additional inserted residues (residues 476-484 in Vip3Bc1 and 10 residues longer in Vip3Ba1 and Vip3Bb1, Supplementary Fig. 4). Deletion of this insert was one of several mutations introduced in order to elucidate the only existing Vip3B structure, mutant VipB2160^15^. In Vip3Bc1 the additional sequence is part of region containing one of the two short alpha helices in domain 3, which sit exposed in the tetrameric assembly.

Domains 4 and 5 of Vip3Bc1 bear striking structural similarity to each other, with superposition yielding an RMSD of 5.9 Å despite low sequence identity (22%), with a twisted beta-sheet ‘jelly roll’ fold that they share with domain 3 of the 3D-Cry proteins (Fig. 4). Domain 3 of 3D-Cry proteins has been implicated in target specificity and glycan binding in previous studies^24^. The role of Vip3 domains 4 and 5 in target insect specificity may be supported by other studies that showed mutation of residues in domain 4 of Vip3Aa11 altered toxicity levels against *Spodoptera exigua* and *Helicoverpa armigera*^25^ and alanine scanning mutagenesis of the Vip3Af1 protein identified a number of mutants in this region that caused significant loss of activity against *Spodoptera frugiperda* and *Agrotis segetum*^26^. Furthermore, DALI analysis of domains 4 and 5 of Vip3Bc1 identified a number of structural homologues of these domains with putative and demonstrated glycan binding capabilities^27^. It is therefore reasonable to suggest that these domains may play a role in receptor engagement.

### Structural variation in the Vip3 family

Several structures have been released for Vip3 variants recently^15,16,18^. Structures of the unprocessed Vip3, including that for Vip3Bc1 presented here, show the same overarching morphology in their bioassembly (Supplementary Fig. 7). When inspected at the primary sequence level, Vip3 variants share a high degree of sequence identity, and consequently structural identity, particularly in domains 1 and 2 which are involved in proteolytic activation, and the global conformational change observed between Vip3Bc1 and Vip3Bc1^act^. We can use the recent findings to propose that all Vip3 share a common mechanism of activation and action.

However, subtle structural differences are observed between Vip3 structures available to date. Domains 3–5 show a much higher degree of sequence divergence across the Vip3 family, which is reflected in differing relative orientations of domains 3, 4 and 5 when comparing Vip3Bc1 and Vip3Aa16 (Supplementary Fig. 7). In the Vip3Aa16 structure, loops on domain 5 appear to dock into pockets in domain 3, with interacting residues well conserved within Vip3A but not within Vip3B and C. In addition, Vip3Bc1 domain 3 has extended insert sequences (Supplementary Fig. 4). This leaves domain 5 to occupy a distinct orientation relative to domain 3 in Vip3Bc1 vs Vip3Aa16. Structural variations in domains 3–5 might underpin the difference in target species specificity that we see amongst different Vip3 toxins. These recent structural insights provide the basis for interesting future experiments to tailor toxin specificity.

### Activation and membrane interaction

**Fig. 5:**
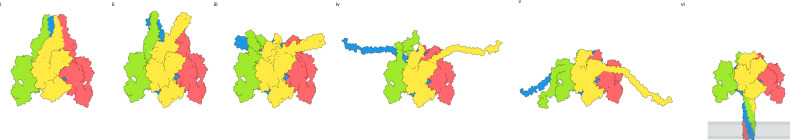
Proposed model of Vip3Bc1 conformational rearrangement upon processing. Cleavage at K205 liberates helices α1-α4 allowing dissociation of inter-subunit interactions at the tip of the complex (i), the helices of domains 2 that are C-terminal to the cut site (from α5 onwards), remain in essentially their original position stabilising the central interface of the tetramer. The α4 helices form a central helical bundle while the other helices N-terminal to the cut site (α1-3) are able to unfurl and move down between the propeller domains to form a new helical bundle (ii–v), which interacts with the membrane via the ends of the helical bundle (vi).

Bringing structural and biochemical data together, we propose a model for this structural rearrangement and mechanism of action in the context of the biological assembly (Fig. 5). After cleavage at K205 the remaining helices of domain 2 that are C-terminal to the cut site (from helix α5 onwards), remain in essentially their original position stabilising the central interface of the tetramer. The dramatic changes induced by proteolysis arise from the liberation of residues N-terminal to the cleavage site, and we propose this conformational change cannot occur in the absence of cleavage on the K205 loop. Following cleavage, inter-subunit interactions at the conical tip of the complex dissociate, allowing for helices α1–α3 to rotate and move down through the gap between the ‘propeller’ regions of adjacent monomers, and form the extended helical bundle at the base of the complex, as visualised via cryoET (Fig. 3) and consistent with the reports for Vip3Aa16^16^. The tips of this helical bundle may then insert into the membrane, and in doing so form a pore. The segmented cryoET data illustrates that the model built for Vip3Bc1^act^ does not have a sufficiently long alpha-helical bundle to fully accommodate the cryoET density. While models for other Vip3 variants^16^ have proposed longest dimension of the activated complex to be ~24 nm, in the cryoET data presented here we only see ~15 nm of density outside of the membrane. This supports the view that we are observing Vip3Bc1^act^ inserting into the membrane. The helical bundle forms a ~5 Å pore, which we propose acts to shuttle small ions following membrane insertion, and therefore imparts the membrane perturbing activity of Vip3Bc1.

The liberated helices α1-3 (Fig. 5) are structurally mobile and not observed in our Vip3Bc1^act^ structure. We propose that, at least in Vip3Bc1, these helices are relatively flexible in solution. However, our cryoET data clearly show a stalk (Fig. 3) consistent with a conformational change of Vip3Bc1, in which these helices form a membrane pore. Density for the stalk was also observed in the Vip3Aa structure. This may indicate that, in solution, the Vip3Aa16 stalk is more stable than that of Vip3Bc1, or alternatively the continuous carbon surface on the cryoEM grid may have stabilised the stalk region of Vip3Aa16^16^.

Image analysis of the Vip3Bc1 and Vip3Bc1^act^ revealed heterogeneity, with populations of both structural conformers present in all datasets. We were able to classify a subset of particles from the Vip3Bc1 dataset into a 3D class corresponding to the activated conformation (Supplementary Fig. 2). For Vip3Bc1^act^, SDS-PAGE gel analysis indicates almost complete digestion of the complexes (Supplementary Fig. 1), but ~16% of particles in the Vip3Bct^act^ population classified into a 3D structure corresponding to Vip3Bc1 (Supplementary Fig. 6). This could represent tetrameric complex where one or more of the monomers remains unprocessed, which may hold the partially digested complex in the Vip3Bc1 conformation. However other factors may also contribute to the initiation of the conformational change, as in the case of Vip3Aa35 ~30% of proteolytically activated protein was reported to remain in the inactivated conformation despite >95% processing reported.

Particles adopting Vip3Bc1^act^ -like conformation were also found in the Vip3Bc1 dataset (Supplementary Fig. 2). This suggests (i) that cleavage on the loop containing K205 is not needed for conformational change or (ii) that between SDS PAGE analysis and imaging by cryoEM, cleavage occurred, enabling the conformational change. While this remains an unexplained observation, based on the structures available we believe that cleavage on the K205 loop is required for conformational change to take place, given the large steric clashes that would result if the conformational change was attempted without cleavage of all four monomers.

Significantly, in our cryoET data we are clearly visualising the Vip3Bc1^act^ interacting with the membrane via the stalk region, and we propose that this shows the physiologically relevant pore conformation. Comparisons of tomograms of Vip3Bc1 show a large variation in the number of protein molecules observed bound to the membrane. The majority of LUVs imaged did not have protein bound, while a small number of LUVs were highly enriched in Vip3Bc1 (Supplementary Movie 2). This finding suggests that Vip3Bc1^act^ particles likely bind to the first membrane they encounter in solution, in a diffusion limited manner. When taken together with the liposome dye release assay results, these data suggest that receptor engagement is not necessary for activated Vip3 to insert into membranes and impart its membrane perturbing toxicity. This phenomenon is also observed with other pore-forming proteins, including 3D-Cry toxins, which can form pores in artificial membranes independent of a receptor^28–30^. However, the in situ activity of activated Vip3 on the biomembrane may also be influenced by factors including lipid composition, integral or membrane associated proteins and glycosylation. We note that the concentration of toxin present in our experiments likely does not reflect that present in the gut of target insects—where receptor engagement likely acts to capture toxin at the membrane thereby increasing the effective concentration. Existing structural data do not illuminate whether receptor engagement happens before or after processing, indeed receptor engagement may occur in both conformations. Further studies are required to shed light on this area.

The dimensions of the complex observed on the membrane via cryoET indicate that only a small portion of the extended helical stalk would insert into the membrane. This is reminiscent of the proposed activity of other alpha-helical pore forming toxins such as the YaxAB toxins^31^ and HlyE (also known as ClyA^32^.

Previous biochemical analysis has suggested that Vip3Aa complex forms a pore in membranes of 14 Å in a planar lipid bilayer at pH 8.0^10^, consistent with structures of activated Vip3 presented here and elsewhere. The proposal that domains 1 and 2 of Vip3 proteins are involved in pore formation is supported by alanine scanning mutagenesis experiments that highlighted a number of mutants clustered in amino acids 167–272 of the Vip3Af1 protein (a domain 2 region equivalent to residues 177–282 of Vip3Bc1) that caused significant loss of activity against *S. frugiperda* and *A. segetum*^26^. We would propose that these mutants may interfere with the pore forming role of domain 2.

Several important outstanding questions remain about how Vip3 exerts its cytotoxicity in situ. Vip3Bc1 is thought to exert its toxic effect in the lepidopteran midgut, a high pH (>pH 10) environment^33^, however here we demonstrate that Vip3Bc1^act^ is highly active at pH 8.5, and previous biochemical studies show Vip3Aa35 is inactive (and aggregates) at pH 10 (ref. ^10^). Taking these data together, we suggest Vip3 toxins must be processed and exert their pore forming action in a more pH neutral environment than that of the high pH midgut lumen. Two possibilities may allow for this, firstly that regional microenvironments exist within the midgut and allow pore formation to occur. Alternatively, the Vip3 tetrameric complex may be internalised to intracellular compartments before exerting its cytotoxic effects. The generation of neoepitope antibodies or other protein conformation specific binders that can distinguish between Vip3 and Vip3^act^ could be important tools to help address these questions in a cellular context.

In summary, our study gives insight into the mechanism by which activated Vip3 complexes perturb membranes. Further analysis of the Vip3 family of toxins will be required to shed light on their cytotoxicity in situ, and on the role of receptor engagement in species specificity of different Vip3 variants.

## Methods

### Purification of Vip3Bc1

The Vip3Bc1 protein was expressed in *Pseudomonas fluorescens* and purified as previously described^9^. For undigested Vip3Bc1, the protein (1 mg/mL) was loaded onto a pre-equilibrated Superose 6 increase 10/300 GL column (25 mM TrisHCl pH 7.5 and 150 mM NaCl). For the trypsin digest, Vip3Bc1 (1 mg/mL, 25 mM TrisHCl pH 8 150 mM NaCl) was incubated with trypsin (1:100 molar ratio) and incubated at 37 °C for 1 h. The sample was then loaded onto a pre-equilibrated Superose 6 increase 10/300 GL column with 25 mM TrisHCl pH 7.5 and 150 mM NaCl.

### CryoEM grid preparation

Protein for single particle analysis was prepared using Quantifoil R 1.2/1.3 Cu 300 grids, glow-discharged in air using the Quorum GloQube^®^ for 30 s at 40 mA (Vip3Bc1) or plasma cleaned using a Tergeo-EM plasma cleaner (Pie Scientific) (Vip3Bc1^act^). 3 µL of sample was applied to the grids in a Vitrobot Mark IV (Thermo Fisher Scientific) at 95% relative humidity, 4 °C, blotted for 6 s with a blot force of ‘6’ and plunged into liquid ethane.

For grids containing LUVs, samples were vitrified ~6 h after LUV and protein were mixed. LUV solution was mixed 2:1 with a concentrated stock of 10 nm fiducial markers resuspended in the same buffer as LUVs. Quantifoil R 2/2 Cu 200 grids were glow-discharged in air in a Quorum GloQube^®^ for 30 s at 40 mA prior cryoEM grid preparation. 3 µL of sample was applied to the grids in a Vitrobot Mark IV (Thermo Fisher Scientific) at 95% relative humidity, 4 °C, blotted for 6 s with a blot force of ‘6’ and plunged into liquid ethane.

### CryoEM imaging

Single particle cryoEM data were collected on a Titan Krios microscope (‘Titan Krios 1’ at the Astbury Biostructure Laboratory) operating at 300 kV and Falcon III direct electron detector (Thermo Fisher Scientific) operating in integrating mode. Data were collected using EPU software with parameters as in Supplementary Table 1, based on a published protocol^34^. Where the sample was tilted during collection, the autofocus and drift measurements were taken in line with the tilt axis.

Tilt series data were collected using a Titan Krios microscope (‘Titan Krios 2’) operating at 300 kV and Bioquantum energy filter (20 eV) K2 direct electron detector (Gatan) operating in counting mode, using Tomo software (Thermo Fisher Scientific). Data were collected with parameters as in Supplementary Table 2.

### Single particle cryoEM data processing

All frames of each micrograph movie were motion-corrected, dose weighted, and merged using Motioncor2^35^. The contrast transfer function (CTF) for each micrograph was determined using Gctf^36^ on motion-corrected, but non-dose weighted, micrographs. Particles were selected with crYOLO^37^ using the PhosaurusNet general model. Individual particles were extracted into 264 × 264 pixel (281 Å) boxes for Vip3Bc1 or 300 × 300 pixel (320 Å) boxes for Vip3Bc1 and culled with multiple rounds of 2D classification in Relion 3^38^. Asymmetric starting models were created in Relion 3 using stochastic gradient descent (SGD)^39^ and manually aligned on the C2/C4 symmetry axes using UCSF Chimera^40^. The particles were further culled by multiple rounds of 3D classification (heterogeneous refinement) in cryoSPARC. Heterogenous refinements for Vip3Bc1 were performed first with two copies of the SGD model as references, and subsequently with two copies of the best result from the previous reconstruction in later iterations. Heterogenous refinements for Vip3Bc1 were first performed with three copies of the SGD model as references, followed by two copies of the previous best result and one copy of the undigested Vip3Bc1 to remove images of undigested particles, and finally two copies of the best result from the previous iteration. All reference models were filtered to 20 Å and heterogenous reconstructions performed with C2 symmetry applied. Final reconstructions were performed in cryoSPARC using non-uniform refinement for Vip3Bc1 (C2 symmetry applied) and homogenous refinement for Vip3Bc1 (C4 symmetry applied). (Supplementary Fig. 2). 3DFSC were calculated using Remote 3DFSC^19^.

### Classification for determination of proportion of cleaved particles in the untreated dataset

The 957,014 particles generated from the Vip3Bc1 2D classification were subjected to one round of heterogenous refinement in cryoSPARC using two copies of the starting model generated by SGD. One of the two starting models was unambiguously of the complex Vip3Bc1 whilst the other was unclear. The ambiguous model was subject to an additional round of heterogenous refinement in cryoSPARC using two copies of the Vip3Bc1 and one copy of Vip3Bc1 generated from the trypsin-treated sample. This resulted in three classes: one, which contained ~31% of the original particles were unambiguously the cleaved Vip3Bc1 (Supplementary Fig. 6). All refinements were performed with C1 symmetry.

### Tilt series data processing

Image processing was carried out identically for all tomograms presented. Micrograph movies were frames of each micrograph movie were motion-corrected and merged using Motioncor2. Tilt series were processed and constructed into tomograms using fiducial-less alignment in IMOD (bin2)^41^. Reconstructed tomograms were filtered in Fiji^42^ using a 3D gaussian (2 sigma) filter and adjusted for brightness and contrast to aid visualisation. Tomograms were segmented using Amira (Thermo Fisher Scientific), using the interactive thresholding tool to segment density.

### Model building

The Vip3Bc1 pre-processed map was of sufficient quality to allow for secondary structure assignment to the core helical domain (domain 1). Initial secondary structure fragments were generated using Bucaneer and extended manually in COOT (residues 27–398). The density for domains 2–4 of Vip3Bc1 were of insufficient resolution to allow for de novo model building, for this portion of the model, domains 2–4 from PDB 6V1V were used to generate homology models of Vip3Bc1. Homology modelling was carried out with the SWISS model server. The resultant models were rigid body fitted into the cryoEM map using UCSF chimera and showed good agreement with experimental map. Local fitting of residues and connectivity of domains was carried out in COOT. The register of amino acid sequence for Vip3Bc1 was cross referenced with that of PDB 6V16 to ensure correct assignment in lower resolution areas of the map.

The Vip3Bc1^act^ map was not of sufficient resolution to allow for de novo model building. Instead residues 105-336 corresponding to domains 1 and 2 were modelled by homology to Vip3Aa (pdb:6tfk) using the SWISS model server. Residues 337-803, corresponding to domains 3,4 and 5, from the Vip3Bc1 (pre-toxin) model generated in this study were attached to residues 105-336 using COOT in order to generate a complete model for Vip3Bc1^act^. This model is deposited in the PDB as 6YRG. A second model for Vip3Bc1^act^ was prepared that describes only residues visible in the experimental map. This excludes residues 105–140, which appear to be disordered in the specimen used here. The second model is deposited in the PDB as 7NTX.

### Liquid chromatography-mass spectrometry

Protein desalting and mass analysis was performed by LC-MS using an M-class ACQUITY UPLC (Waters UK, Manchester, UK) interfaced to a Xevo QToF G2-XS mass spectrometer (Waters UK, Manchester, UK). Samples were diluted to 5 µM using 0.1% TFA. 1 µL of the 5 µM sample was loaded onto a MassPREP protein desalting column (Waters UK, Manchester, UK) washed with 10% solvent B in A for 5 min at 25 µL min. After valve switching, the bound protein was eluted by a gradient of 2–40% solvent B in A over 1 min at 25 µL min. The column was subsequently washed with 95% solvent B in A for 6 min before re-equilibration at 5% solvent B in A ready for the next injection. Solvent A was 0.1% formic acid in water, solvent B was 0.1% formic acid in acetonitrile.

The column eluent was directed into the mass spectrometer via a Z-spray electrospray source. The MS was operated in positive TOF mode using a capillary voltage of 3.2 kV, sample cone of 20 V and source offset of 80 V. The source temperature was 100 °C and desolvation was 250 °C. Mass calibration was performed by a separate injection of [Glu]-fibrinopeptide b at a concentration of 250 fmol µL. Data processing was performed using the MassLynx v4.1 suite of software supplied with the mass spectrometer.

### Preparation of LUVs

CF-loaded LUVs were prepared from a lipid mixture of DOPC and DOPE in a molar ratio 6:4 dissolved in chloroform. First, 100 µL of the lipid solution were dried under high vacuum overnight to get a dry lipid thin film. 5(6)-CF was encapsulated within LUVs at sufficient concentration to be self-quenched by rehydration of the lipid film with 500 µL of 50 mM TrisHCl, 120 mM CF at pH 8.5 to form a suspension of polydisperse multilamellar liposomes. This liposome suspension was then subjected to 5 freeze-thaw cycles and extruded 11 times through a 400 nm pore size polycarbonate membrane (Whatman International Ltd) using a LiposoFast liposome extruder (Avestin Inc). The final suspension of monodisperse LUVs was passed through a Sephadex G-25 column to remove the unencapsulated CF via SEC.

### Determination of lipid concentration

The phospholipid concentration in the LUVs sample was determined by a standard phosphorus assay. Briefly, 70 μL aliquots of the vesicle sample were added to sample test tubes and calibration samples were created using a phosphorous standard solution (0, 0.0325, 0.065, 0.114, 0.163, 0.228 μM phosphorous). 450 μL 8.9 N H_2_SO_4_ (aq) were added to each test tube and heated to 215 °C for 25 min. After cooling the samples at room temperature, 150 μL H_2_O_2_ (30% w/v) were added and the tubes were heated again at 215 °C for 30 min. Test tubes were allowed to cool before adding 3.9 mL deionised water, 0.5 mL 2.5% ammonium molybdate (VI) tetrahydrate solution and 0.5 mL 10% ascorbic acid solution then heating at 100 °C for 7 min. Finally, the adsorption of each sample was measured at 820 nm and the concentration of phosphorous (and therefore phospholipid) in the LUVs sample was determined by comparison to the calibration curve created using the phosphorous standards.

### 5(6)-Carboxyfluorescein release assay

CF-loaded LUVs were suspended in isotonic buffer (50 mM TrisHCl, 150 mM NaCl, pH 8.5) reaching a lipid concentration of 2.0 ± 0.5 µM and incubated for 30 min with different concentrations of Vip3Bc1 before and after trypsin treatment. The CF release from the liposomes was detected by measuring the fluorescence intensity from 500 nm to 600 nm of the samples excited at 492 nm, using a FluoroMax-Plus spectrofluorimeter (Horiba Scientific). Three independent replicates were taken for each data point shown in Fig. 2. The results are presented as normalised percentage of dye release calculated from the fluorescence emission peaks at 514 nm (*I*_*n*_). Samples of LUVs non-exposed to protein were used as baseline signal (*I*_*0*_) and values for 100% dye release (*I*_*max*_) were obtained by lysing the LUVs with 0.15% (w/v) of Triton X-100. The normalised fraction of CF release (*L*_*n*_) is given by:

$${L}_{n}=\frac{{I}_{n}-{I}_{0}}{{I}_{\max}-{I}_{0}}x1$$

## Supplementary information

Supplementary Information

Supplementary Movie 1

Supplementary Movie 2

Supplementary Movie 3

Supplementary Movie 4

## Data Availability

Data supporting the findings of this paper are available from the corresponding authors upon reasonable request. A reporting summary for this Article is available as a Supplementary Information file. EM density maps have been deposited in the Electron Microscopy Data Bank under accession number EMD-10888 for Vip3bc1 and EMD-10889 for Vip3Bc1^act^ and atomic coordinates into the Protein Data Bank under accession number 6YRF for Vip3bc1 and 6YRG and 7NTX for Vip3Bc1^act^. Source data are provided with this paper.

## Source data

Source Data
