# Supplementary material for: Cryo-EM structures of an insecticidal Bt toxin reveal its mechanism of action on the membrane
Source: Nat Commun. 2021 May 14;12:2791. doi: 10.1038/s41467-021-23146-4 (PMC8121907; doi:10.1038/s41467-021-23146-4)
Supplement: Supplementary file 1 — Supplementary Information [file 41467_2021_23146_MOESM1_ESM.pdf]

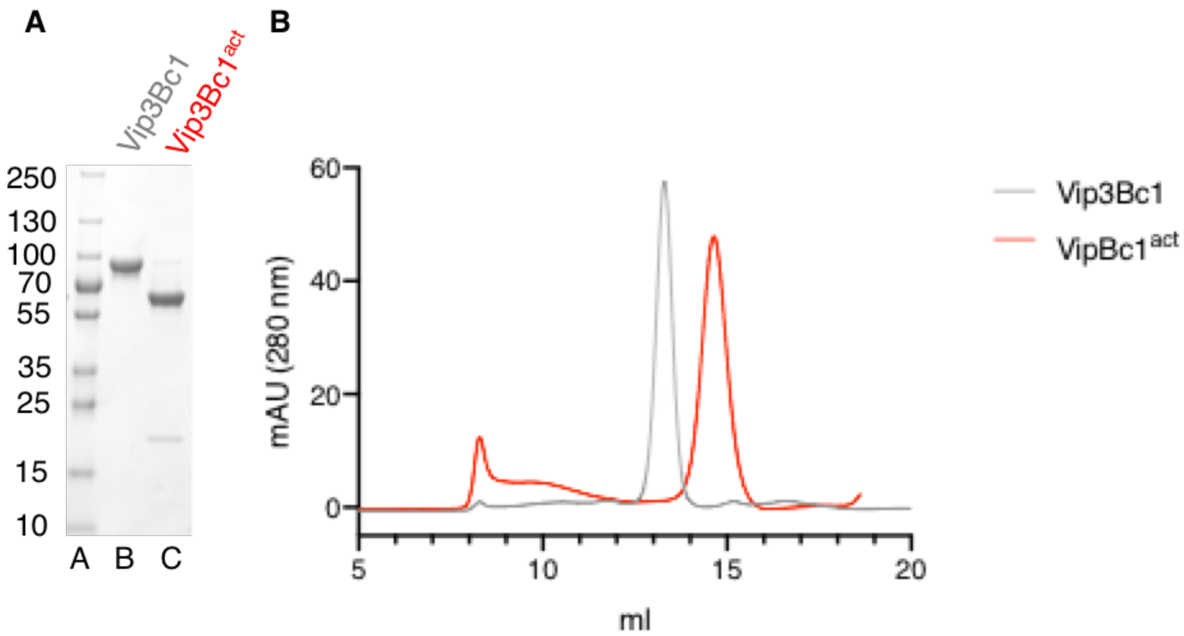


**Supplementary Figure 1- Trypsin digestion and SEC analysis of Vip3Bc1.** A) SDS-PAGE analysis of Vip3Bc1 (lane B) and Vip3Bc1$^{act}$ (lane C) protein with molecular weight marker (kDa) (lane A). B) Elution from size exclusion chromatography (SEC) for Vip3Bc1 (grey line) and trypsin treated Vip3Bc1$^{act}$ (Red line). Upon trypsin treatment, Vip3Bc1$^{act}$ shows a shift in mass via both SDS-PAGE and SEC. Source data are provided as a source data file.

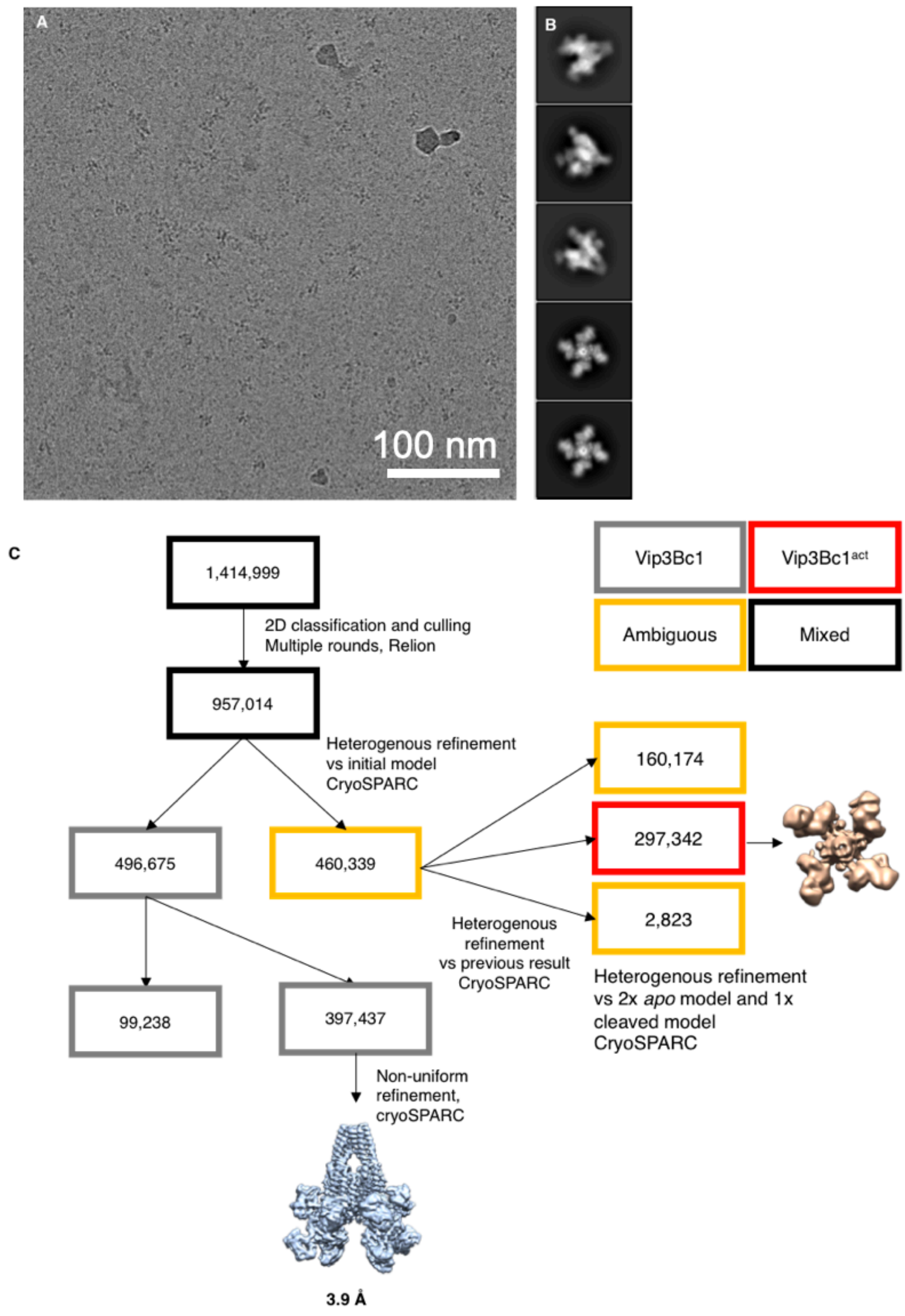


**Supplementary Figure 2- Image processing of Vip3Bc1 dataset.** A) Representative micrograph from a dataset of 17900 images and B) representative 2D class averages showing a range of angular orientations. C) Image processing pipelines for Vip3Bc1. Numbers indicate #particles at given stages during the reconstruction process. Reconstruction statistics can be seen in Supplementary table 1.

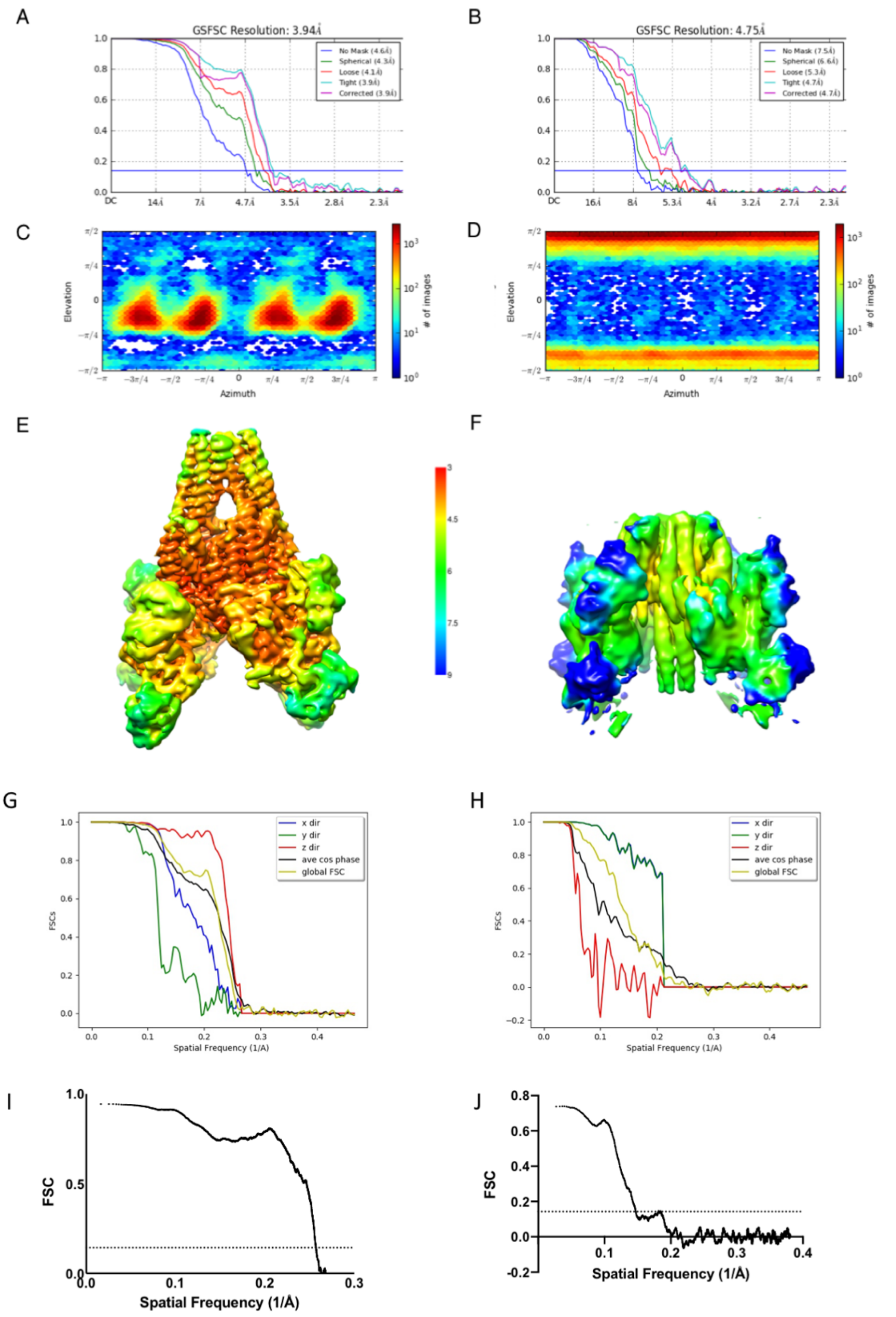


**Supplementary Figure 3- Evaluation of Vip3Bc1 cryoEM density maps**. Fourier shell correlation (FSC) curves for Vip3Bc1 (A) and Vip3Bc1$^{act}$ (B) reconstructions, and corresponding angular distribution maps for Vip3Bc1 (C) and Vip3Bc1$^{act}$ (D), showing Vip3Bc1$^{act}$ has a strong preferred orientation. EM density coloured for local resolution for Vip3Bc1 (E) and Vip3Bc1$^{act}$ (F) reconstructions show local resolution variations (colour bar indicates local resolution in Å). 3D FSC curves for Vip3Bc1 (G) and Vip3Bc1$^{act}$ (H). Model Map FSC (dotted line on Y axis 0.143 FSC) for Vip3Bc1 (I) and Vip3Bc1$^{act}$ (J).

```
Vip3Bc1             MVQKWMQRMIIVDNNKLNVRALPSFIDYFNGIYGFATGIKDIMGMIFKTDTGGSNLTLDE 60
Vip3B2160      MGSSHHHHHHQRMIIVDNNKLNVRALPSFIDYFNGIYGFATGIKDIMGMIFKTDTGGSNLTLDE 55
Vip3Ba1                      MNMNNTKLNARALPSFIDYFNGIYGFATGIKDIMNMIFKTDTGGGNLTLDE 51
Vip3Bb1                        MNNTKLNARALPSFIDYFNGIYGFATGIKDIMNMIFKTDTGGGNLTLDE 49
Vip3Ca1                      MNKNNTKLNARALPSFIDYFNGIYGFATGIKDIMNMIFKTDTGG-DLTLDE 50
Vip3Ah1                      MNKNNTKLSTRALPSFIDYFNGIYGFATGIKDIMNMIFKTDTGG-DLTLDE 50
Vip3Aa1                      MNKNNTKLSTRALPSFIDYFNGIYGFATGIKDIMNMIFKTDTGG-DLTLDE 50
Vip3Aa16                     MNKNNTKLSTRALPSFIDYFNGIYGFATGIKDIMNMIFKTDTGG-DLTLDE 50
Vip3Af1                      MNMNNTKLNARALPSFIDYFNGIYGFATGIKDIMNMIFKTDTGG-NLTLDE 50
Vip3Ai1                    MNMNKNNTKLSARALPSFIDYFNGIYGFATGIKDIMNMIFKTDTGG-DLTLDE 52
Vip3Ac1                       MQKNNKLSVKALPSFIDYFNGIYGFATGIKDIMNMIFKTNTGG-DLTLDE 49
Vip3Ad1                      MNMNNTKLNARALPSFIDYFNGIYGFATGIKDIMNMIFKTDTGG-NLTLDE 50
Vip3Ab1                      MNMNNTKLNARALPSFIDYFNGIYGFATGIKDIMNMIFKTDTGG-NLTLDE 50
Vip3Aj1                      MNKNNTKLNARALPSFIDYFNGIYGFAIGIKDIMNMIFKTDTGS-NLTLDE 50
Vip3Ae1                      MNMNNTKLNARALPSFIDYFNGIYGFATGIKDIMNMIFKTDTGG-NLTLDE 50
Vip3Ag1                      MNKNNTKLNARALPSFIDYFNGIYGFATGIKDIMNMIFKTDTGG-NLTLDE 50
                                .*.**..:******************** ******.*****:**. :******

Vip3Bc1             ILKNQNLLNDISGKLDGINGDLGDLIAQGNLNSELAKELLKISNEQNQMLNHVNAQLNAI 120
Vip3B2160           ILKNQNLLNDISGKLDGINGDLGDLIAQGNLNSELAKELLKISNEQNQMLNHVNAQLNAI 115
Vip3Ba1             ILKNQDLLNQISDKLDGINGDLGDLIAQGNLNSELTKELLKIANEQNLMLNNVNAQLNSI 111
Vip3Bb1             ILKNQDLLNQISDKLDGINGDLGDLIAQGNLNSELTKELLKIANEQNLMLNNVNAQLNSI 109
Vip3Ca1             ILKNQQLLNEISGKLDGVNGSLNDLIAQGNLNTELSKEILKIANEQNQVLNDVNNKLDAI 110
Vip3Ah1             ILKNQQLLNEISGKLDGVNGSLNDLIAQGNLNTELSKEILKIANEQNQVLNDVNNKLDAI 110
Vip3Aa1             ILKNQQLLNDISGKLDGVNGSLNDLIAQGNLNTELSKEILKIANEQNQVLNDVNNKLDAI 110
Vip3Aa16            ILKNQQLLNDISGKLDGVNGSLNDLIAQGNLNTELSKEILKIANEQNQVLNDVNNKLDAI 110
Vip3Af1             ILKNQQLLNEISGKLDGVNGSLNDLIAQGNLNTELSKEILKIANEQNQVLNDVNNKLDAI 110
Vip3Ai1             ILKNQQLLNDISGKLDGVNGSLNDLIAQGNLNTELSKEILKIANEQNQVLNDVNNKLDAI 112
Vip3Ac1             ILKNQQLLNEISGKLDGVNGSLNDLLAQGNLDTELSKEILKIANEQNKVLNDVNTKLDAI 109
Vip3Ad1             ILKNQQLLNEISGKLDGVNGSLNDLIAQGNLNTELAKQILKVANEQNQVLNDVNNKLDAI 110
Vip3Ab1             ILKNQQLLNEISGKLDGVNGSLNDLIAQGNLNTELSKEILKIANEQNQVLNDVNNKLDAI 110
Vip3Aj1             ILKNQQLLNEISGKLDGVNGSLNDLIAQGNLNTELAKQILKVANEQNQVLNDVNNKLDTI 110
Vip3Ae1             ILKNQQLLNEISGKLDGVNGSLNDLIAQGNLNTELSKEILKIANEQNQVLNDVNNKLDAI 110
Vip3Ag1             ILKNQQLLNEISGKLDGVNGSLNDLIAQGNLNTELSKEILKIANEQNQVLNDVNNKLNAI 110
                    *****:***:**.****:**.*.**:*****::**:*::**::**** :**.** :*::*

Vip3Bc1             NSTLNIYLPKITSMLNEVMKQNHVLSLQIEFLSKQLQEISDKLDIINLNVLINSTLTEIT 180
Vip3B2160           NSTLNIYLPKITSMLNEVMKQNHVLSLQIEFLSKQLQEISDKLDIINLNVLINSTLTEIT 175
Vip3Ba1             NATLNTYLPKITSMLNEVMKQNYVLSLQIEFLSKQLQEISDKLDIINLNVLINSTLTEIT 171
Vip3Bb1             NSTLNTYLPKITSMLSEVMKQNYVLSLQIEFLSEQLQEISDKLDVINLNVLINSTLTEIT 169
Vip3Ca1             NTMLNIYLPKITSMLSDVMKQNYALSLQIEYLSRQLQEISDKLDVINLNVLINSTLTEIT 170
Vip3Ah1             NTMLHIYLPKITSMLSDVMKQNYALSLQIEYLSKQLQEISDKLDIINVNVLINSTLTEIT 170
Vip3Aa1             NTMLRVYLPKITSMLSDVMKQNYALSLQIEYLSKQLQEISDKLDIINVNVLINSTLTEIT 170
Vip3Aa16            NTMLRVYLPKLTSMLSDVMKQNYALSLQIEYLSKQLQEISDKLDIINVNVLINSTLTEIT 170
Vip3Af1             NTMLHIYLPKITSMLSDVMKQNYALSLQIEYLSKQLQEISDKLDIINVNVLINSTLTEIT 170
Vip3Ai1             NTMLHVYLPKITSMLSDVMKPNYALSMQIEYLSRQLQEISDKLDIINVNVLINSTLTEIT 172
Vip3Ac1             NLMLNTYLPKITSMLSDVMKQNYALGLQIEYLSKQLKEISDKLDVINVNVLINSTLTEIT 169
Vip3Ad1             NSMLKIYLPKITSMLSDVMKQNYVLSLQIEYLSKQLQEISDKLDIINVNVLINSTLTEIT 170
Vip3Ab1             NTMLHIYLPKITSMLSDVMKQNYALSLQVEYLSKQLKEISDKLDVINVNVLINSTLTEIT 170
Vip3Aj1             NSMLKIYLPKITSMLSDVMKQNYVLSLQIEYLSKQLQEISDKLDIINVNVLINSTLTEIT 170
Vip3Ae1             NTMLHIYLPKITSMLSDVMKQNYALSLQIEYLSKQLQEISDKLDIINVNVLINSTLTEIT 170
Vip3Ag1             NTMLHIYLPKITSMLNDVMKQNYALSLQIEYLSKQLQEISDKLDVINVNVLINSTLTEIT 170
                    *  *. ****:****.:*** *:.*.:*:*:**.**:*******:**:************
```

```
                                        K205
                                          |
Vip3Bc1      PAYQRIKYVNEKFDELTSTVEKNPKSYQD-----NVTKEVIENLNELTELAKSVTKNDMD 235
Vip3B2160    PAYQRIKYVNEKFDELTSTVEKNPKSYQD-----NVTKEVIENLNELTELAKSVTKNDMD 230
Vip3Ba1      PAYQRIKYVNDKFDELTSTVEKNPKINQD-----NFTEDVIDNLTDLTELARSVTRNDMD 226
Vip3Bb1      PAYQRIKYVNDKFDELTSTVEKNPKINQD-----NFTEDVIDNLTDLTELARSVTRNDMD 224
Vip3Ca1      PSYQRIKYVNEKFDKLTFATESTLRAKQGIFNEDSFDNNTLENLTDLAELAKSITKNDVD 230
Vip3Ah1      PAYQRIKYVNEKFEELTFATETNLKVKKD-----GSPADILDELTELTELAKSVTKNDVD 225
Vip3Aa1      PAYQRIKYVNEKFEELTFATETSSKVKKD-----GSPADILDELTELTELAKSVTKNDVD 225
Vip3Aa16     PAYQRIKYVNEKFEELTFATETSSKVKKD-----GSPADILDELTELTELAKSVTKNDVD 225
Vip3Af1      PAYQRIKYVNEKFEELTFATETTLKVKKD-----SSPADILDELTELTELAKSVTKNDVD 225
Vip3Ai1      PAYQWIKYVNEKFEELTFATETTLKVKND-----SASADILDELTELTELAKSVTKNDVD 227
Vip3Ac1      PAYQRIKYVNEKFEALTSATETNLKTKQD-----SSHTDILDELTELTELAKSVTKNDVD 224
Vip3Ad1      PAYQRIKYVNEKFDDLTFATENTLKVKKD-----SSPADILDELTELTELAKSVTKNDVD 225
Vip3Ab1      PAYQRIKYVNEKFEELTFATETTLKVKKD-----SSPADILDELTELTELAKSVTKNDVD 225
Vip3Aj1      PAYQRMKYVNEKFEELTFATETTLKVKKD-----SPPADILDELTELTELAKSVTKNDVD 225
Vip3Ae1      PAYQRIKYVNEKFEELTFATETTLKVKED-----GSPADILDELTELTELAKSVTKNEVD 225
Vip3Ag1      PAYQRMKYVNEKFEDLTFATETTLKVKKN-----SSPADILDELTELTELAKSVTKNDVD 225
             *:** :****:**: ** :.*.. :  :.     .   : :::*.:*:***:*:*:*::*

Vip3Bc1      SFEFYLQTFHDVMTGNNLFGRSALKTASELITKENVTTRGSEIGKVYNFLIVLTSLQAKA 295
Vip3B2160    SFEFYLQTFHDVMTGNNLFGRSALKTASELITKENVTTRGSEIGKVYNFLIVLTSLQAKA 290
Vip3Ba1      SFEFYIKTFHDVMIGNNLFSRSALKTASELIAKENIHTRGSEIGNVYTFMIVLTSLQAKA 286
Vip3Bb1      SFEFYIKTFHDVMIGNNLFSRSALKTASELIAKENIHTMGSEIGNVYTFMVVLTSLQAKA 284
Vip3Ca1      SFEFYLHTFHDVLIGNNLFGRSALKTASELITKDEIKTSGSEIGKVYSFLIVLTSLQAKA 290
Vip3Ah1      GFEFYLNTFHDVMVGNNLFGRSALKTASELITKENVKTSGSEVGNVYNFLIVLTALQAKA 285
Vip3Aa1      GFEFYLNTFHDVMVGNNLFGRSALKTASELITKENVKTSGSEVGNVYNFLIVLTALQAQA 285
Vip3Aa16     GFEFYLNTFHDVMVGNNLFGRSALKTASELITKENVKTSGSEVGNVYNFLIVLTALQAKA 285
Vip3Af1      GFEFYLNTFHDVMVGNNLFGRSALKTASELIAKENVKTSGSEVGNVYNFLIVLTALQAKA 285
Vip3Ai1      GFEFYLNTFHDVMVGNNLFGRSTLKTASELIAKENVKTSGSEVGNVYNFLVVLTALQAKA 287
Vip3Ac1      GFEFYLNTFHDVMIGNNLFGRSALKTASELIAKENLKTSGSEVGNVYNFLIVLTALQAKA 284
Vip3Ad1      GFEFYLNTFHDVMVGNNLFGRSALKTASELIAKENVKTSGSEVGNVYNFLIVLTALQAKA 285
Vip3Ab1      GFEFYLNTFHDVMVGNNLFGRSALKTASELIAKENVKTSGSEVGNVYNFLIVLTALQAKA 285
Vip3Aj1      GFEFYLNTFHDVMVGNNLFGRSALKTASELIAKENVKTSGSEVGNVYNFLIVLTALQAKA 285
Vip3Ae1      GFEFYLNTFHDVMVGNNLFGRSALKTASELIAKENVKTSGSEVGNVYNFLIVLTALQAKA 285
Vip3Ag1      GFEFYLNTFHDVMVGNNLFGRSALKTASELIAKENVKTSGSEVGNVYNFLIVLTALQAKA 285
             .****::*****: *****.**:********:*::: * ***:*:**.*::***:***:*

Vip3Bc1      FLTLTACRKLLGLTDIDYTQIMNHHIDGQKREFRINILPTLSNNFSNPSYSKNRGSDIDD 355
Vip3B2160    FLTLTACRKLLGLTDIDYTQIMNHHIDGQKREFRINILPTLSNNFSNPSYSKNRGSDIDD 350
Vip3Ba1      FLTLTTCRKLLGLADIDYTQIMNENLDREKEEFRLNILPTLSNDFSNPNYTETLGSDLVD 346
Vip3Bb1      FLTLTACRKLLGLTDIDYTQIMNENLNREKEEFRLNILPTLSNDFSNPNYTETLGSDLVD 344
Vip3Ca1      FLTLTTCRKLLGLSDIDYTSIMNEHLNNEKNEFRDNILPALSNKFSNPSYAKTIGSDNYA 350
Vip3Ah1      FLTLTTCRKLLGLADIDYTSIMNEHLNKEKEEFRVNILPTLSNTFSNPNYIKTKGSDEDA 345
Vip3Aa1      FLTLTTCRKLLGLADIDYTSIMNEHLNKEKEEFRVNILPTLSNTFSNPNYAKVKGSDEDA 345
Vip3Aa16     FLTLTTCRKLLGLADIDYTSIMNEHLNKEKEEFRVNILPTLSNTFSNPNYAKVKGSDEDA 345
Vip3Af1      FLTLTTCRKLLGLADIDYTSIMNEHLNKEKEEFRVNILPTLSNTFSNPNYAKVKGSDEDA 345
Vip3Ai1      FLTLTTCRKLLGLADIDYTSIMNEHLNKEKEEFRVNILPILSNTFSNPNYAKVKGSDEDA 347
Vip3Ac1      FLTLTTCRKLLGLADIDYTPIMNEHLNKEKEEFRVNILPTLSNTFSNPNYEKARGSDKDA 344
Vip3Ad1      FLTLTTCRKLLGLADIDYTSIMNEHLNKEKEEFRVNILPTLSNTFSNPNYAKAKGSNEDT 345
Vip3Ab1      FLTLTTCRKLLGLADIDYTSIMNEHLNKEKEEFRVNILPTLSNTFSNPNYAKVKGSDEDA 345
Vip3Aj1      FLTLTTCRKLLGLADIDYTSIMNEHLNKEKEEFRVNILPTLSNTFSNPNYAKAKGSNEDT 345
Vip3Ae1      FLTLTTCRKLLGLADIDYTSIMNEHLNKEKEEFRVNILPTLSNTFSNPNYVKAKGSDRDA 345
Vip3Ag1      FLTLTTCRKLLGLADIDYTFIMNEHLDKEKEEFRVNILPTLSNTFSNPNYAKAKGSNEDA 345
             *****:*******:***** ***.::: :*.*** **** *** ****.* :  **:
```

```
Vip3Bc1         PIVVLEAAPGYALIGFEILNDPLPILKGYQARLKPNYQVDRESMSETIYGDIHKLFCPKQ 415
Vip3B2160       PIVVLEAAPGYALIGFEILNDPLPILKGYQARLKPNYQVDRESMSETIYGDIHKLFCPKQ 410
Vip3Ba1         PIVTLEAEPGYALIGFEILNDPLPVLKVYQAKLKPNYQVDKESIMENIYGNIHKLLCPKQ 406
Vip3Bb1         PIVTLEADPGYALIGFEILNDPLPVLKVYQAKLKPNYQVDKESIMENIYGNIHKLLCPKQ 404
Vip3Ca1         -KVILESEPGYALVGFEIINDPIPVLKAYKAKLKQNYQVDNQSLSEIVYLDIDKLFCPEN 409
Vip3Ah1         -EVIIQAEPGHALVGFEMINDPSPALKVYQAKLKPNYQVDKQSLSETVYGDMDKILCPDK 404
Vip3Aa1         -KMIVEAKPGHALIGFEISNDSITVLKVYEAKLKQNYQVDKDSLSEVIYGDMDKLLCPDQ 404
Vip3Aa16        -KMIVEAKPGHALIGFEISNDSITVLKVYEAKLKQNYQVDKDSLSEVIYGDMDKLLCPDQ 404
Vip3Af1         -KMIVEAKPGHALVGFEMSNDSITVLKVYEAKLKQNYQVDKDSLSEVIYGDTDKLFCPDQ 404
Vip3Ai1         -KMIVEAKPGHALVGFEISNDSMTVLKVYEAKLKQNYQVDKDSLSEVIYGDMDKLLCPDQ 406
Vip3Ac1         -KIIMEAKPGYALVGFEISKDSIAVLKVYQAKLKHNYQIDKDSLSEIVYGDIDKLLCPDQ 403
Vip3Ad1         -KMIVEAKPGYVLVGFEMSNNSITVLKAYQAKLKKDYQIDKDSLSEIIYSDTDKLLCPDQ 404
Vip3Ab1         -KMIVEAKPGHALVGFEISNDSMTVLKVYEAKLKQNYQVDKDSLSEVIYSDMDKLLCPDQ 404
Vip3Aj1         -KMIVEAKPGYVLVGFEMSNDSITVLKAYQAKLKKDYQIDKDSLSEIIYSDTDKLLCPDQ 404
Vip3Ae1         -KMIVEAKPGYALVGFEMSNDSMTVLKAYQAKLKQDYQVDKDSLSEIVYGDMNKLLCPDQ 404
Vip3Ag1         -KIIVEAKPGYALVGFEMSNDSITVLKAYQAKLKQDYQVDKDSLSEIVYGDMDKLLCPDQ 404
                  : ::: **:.*:***: ::    ** *:*:** :**:*.:*: * :* : .*::**.:

Vip3Bc1         LEQKYYIKDIEFPEGYVITKIVFEKRLNQLGYEVTANFYDPSTGSIDLNKVKVESWKEKS 475
Vip3B2160       LEQKYYIKDIEFPEGYVITKIVFEKRLNQLGYEVTANFYDPSTGSIDLNKVKVES----S 466
Vip3Bb1         RHQKYYIKDITFPEGYVITKIVFEKKLNLLGYEVTANLYDPFTGSIDLNKTILESWKEEC 464
Vip3Ca1         SEQKYYTKNLTFPDGYVITKITFEKKLNNLIYEATANFYDPSTGDIDLNKKQVESTFPQT 469
Vip3Ah1         SQQMYYLHNITFPNEYVITEIIFTKKKNSLRYEVIANYYEFSSGDIDLNKKLVKSSEAEY 464
Vip3Aa1         SEQIYYTNNIVFPNEYVITKIDFTKKMKTLRYEVTANFYDSSTGEIDLNKKKVESSEAEY 464
Vip3Aa16        SEQIYYTNNIVFPNEYVITKIDFTKKMKTLRYEVTANFYDSSTGEIDLNKKKVESSEAEY 464
Vip3Af1         SEQIYYTNNIVFPNEYVITKIDFTKKMKTLRYEVTANFYDSSTGEIDLNKKKVESSEAEY 464
Vip3Ai1         SEKIYYTNNIVFPNEYVITKIDFTKKMKTLRYEVTANSYDSSTGEIDLNKKKVESSKAEY 466
Vip3Ac1         SEQMYYTNKIAFPNEYVITKIAFTKKLNSLRYEVTANFYDSSTGDIDLNKKIESSEAEF 463
Vip3Ad1         SEQIYYTKNIAFPNEYVITKIAFTKKMNSLRYEATANFYDSSTGDIDLNKTKVESSEAEY 464
Vip3Ab1         SEQIYYTNNIVFPNEYVITKIDFTKKMKTLRYEVTANSYDSSTGEIDLNKKKVESSEAEY 464
Vip3Aj1         SEQIYYTKNIAFPNEYVITKIAFTKKMNSLRYEATANFYDSSTGDIDLNKTKVESSEAEY 464
Vip3Ae1         SEQIYYTNNIAFPREYVITKLTFTKKMNSLRYEATANFYDSSTGDMDLNKTKVESSEAEY 464
Vip3Ag1         SEQIYYTNNIAFPNEYVITKITFTKKMNSLRYEATANFYDSSTGDIDLNKTKVESSEAEY 464
                 .: ** :.: **  ****:: * *: : * **. ** *:  :*.:****  ::*   .

               476                 484 488 492
                |                   |   |   |
Vip3Bc1         CEED----------SCEDEYSIIKAETDGIYMPLGVVSETFLTPIYGFGLTVDEKNQKIT 525
Vip3B2160       --------------SCEDEYSIIKAETDGIYMPLGVVSETFLTPIYGFGLTVDAANAAIT 520
Vip3Ba1         CEEDCCEEDCCEEDCCEELYKIIEADTNGVYMPLGVISETFLTPIYSFKLIIDEKTKKIS 526
Vip3Bb1         CEEECCEEECCEEECCEELYKIIEADTNGVYMPLGVISETFLTPIYSFKLIIDERTKRIS 524
Vip3Ca1         -----------------DYITMDIGDDDGIYMPLGVISETFLTPINSFGLEVDAKSKTLT 512
Vip3Ah1         -------------------S-TLSVSNDAIYMPLGVISETFLTPIKGFGLTVDESSRLVT 504
Vip3Aa1         -------------------R-TLSANDDGVYMPLGVISETFLTPINGFGLQADENSRLIT 504
Vip3Aa16        -------------------R-TLSANDDGVYMPLGVISETFLTPINGFGLQADENSRLIT 504
Vip3Af1         -------------------R-TLSANDDGVYMPLGVISETFLTPINGFGLQADENSRLIT 504
Vip3Ai1         -------------------R-TLSANNDGVYMPLGVISETFLTPINGFGLQADENSRLIT 506
Vip3Ac1         -------------------S-MLNANNDGVYMPIGTISETFLTPINGFGLVVDENSRLVT 503
Vip3Ad1         -------------------S-MLKASDDEVYMPLGLISETFLNPINGFRLAVDENSRLVT 504
Vip3Ab1         -------------------R-TLSANNDGVYMPLGVISETFLTPINGFGLQADENSRLIT 504
Vip3Aj1         -------------------S-MLKASDDEVYMPLGLISETFLNPINGFRLAVDENSRLVT 504
Vip3Ae1         -------------------S-RLSASNDGVYMPLGLISETFLTPINGFGLVVDENSRLVT 504
Vip3Ag1         -------------------S-TLSASTDGVYMPLGIISETFLTPINGFGIVVDENSKLVN 504
                                         . : :***:* :*****.** .* :  *  .: :.
```

```
Vip3Bc1        LTGKSYLRESLLETDLLNNETYLIASPDGYISSIVENWNITSDNTGSWRANNNNAFVDKA 585
Vip3B2160      LTGKSYLRESLLETDLLNNETYLIASPDGYISSIVENWNITSDNTGSWRANNNNAFVDKA 580
Vip3Ba1        LAGKSYLRESLLATDLVNKETNLIPSPNGFISSIVQNWHITSDNIEPWKANNKNAYVDKT 586
Vip3Bb1        LAGKSYLRESLLATDLVNKDTNLIPSPNGFINSIVENWNITSDNIEPWKANNKNAYVDKT 584
Vip3Ca1        LKCKSYLREYLLESDLKNKETGLIAPPNVFISNVVKNWDIEEDSLEPWVANNKNAYVDNT 572
Vip3Ah1        LTCKSYLREILLATDLSNKATKLIVPPNGFISNLVENGDIEADNIEPWKGNNKNAYVDHT 564
Vip3Aa1        LTCKSYLRELLLATDLSNKETKLIVPPSGFISNIVENGSIEEDNLEPWKANNKNAYVDHT 564
Vip3Aa16       LTCKSYLRELLLATDLSNKETKLIVPPSGFISNIVENGSIEEDNLEPWKANNKNAYVDHT 564
Vip3Af1        LTCKSYLRELLLATDLSNKETKLIVPPSGFISNIVENGSIEEDNLEPWKANNKNAYVDHT 564
Vip3Ai1        LTCKSYLRELLLATDLSNKETKLIVPPNSFISNIVENGSIEEGHLEPWKANNKNAYVDHT 566
Vip3Ac1        LTCKSYLRETLLATDLSNKETKLIVPPNGFISNIVENGNLEGENLEPWKANNKNAYVDHT 563
Vip3Ad1        LTCRSYLRETLLATDLNNKETKLIVPPNVFISNIVENGNIEMDTLEPWKANNENANVDYS 564
Vip3Ab1        LTCKSYLRELLLATDLSNKETKLIVPPISFISNIVENGNLEGENLEPWIANNKNAYVDHT 564
Vip3Aj1        LTCRSYLRETLLATDLNNKETKLIVPPNVFISNIVENGNIEMDTLEPWKANNENANVDYS 564
Vip3Ae1        LTCKSYLREILLATDLSNKETKLIVPPNGFISNIVENGNLEGENLEPWKANNKNAYIDHT 564
Vip3Ag1        LTCKSYLREVLLATDLSNKETKLIVPPIGFISNIVENGNLEGENLEPWKANNKNAYVDHT 564
               *  :***** ** :** *: * **  *  :*..:*:*  :       * .**:** :* :

Vip3Bc1        DTIKGSSSLYTHKDGEFSQFIGNKLKPKTNYVIQYVIKGRPAIYLKNN--KDTLFEDTKN 643
Vip3B2160      ----GSSSLYTHKDGEFSQFIGNKLKPKTNYVIQYVIKGRPAIYLKNN--KDTLFEDTKN 634
Vip3Ba1        DAMVGFSSLYTHKDGEFLQFIGAKLKAKTEYIIQYTVKGNPEVYLKNN--KDICYEDKTN 644
Vip3Bb1        DDMVGFNSLYTHKDGEFLQFIGAKLKAKTEYIIQYTVKGSPEVYLKNN--KGIFYEDTTN 642
Vip3Ca1        GGIERSKALFTQGDGEFSQFIGDKLKPNTDYIIQYTVKGKPAIYLKNKSTGYITYEDTNG 632
Vip3Ah1        GGVNGTKALYTQDDGEFSQFIGDKLKSKTEYIIQYTVKGNTSIYLKDKKNENVIYEDKNN 624
Vip3Aa1        GGVNGTKALYVHKDGGISQFIGDKLKPKTEYVIQYTVKGKPSIHLKDENTGYIHYEDTNN 624
Vip3Aa16       GGVNGTKALYVHKDGGISQFIGDKLKPKTEYVIQYTVKGKPSIHLKDENTGYIHYEDTNN 624
Vip3Af1        GGVNGTKALYVHKDGGFSQFIGDKLKPKTEYVIQYTVKGKPSIHLKDENTGYIHYEDTNN 624
Vip3Ai1        GGVNGTKALYVHEDGGVSQFMGDKLKPKTEYVIQYTVKGKPSIHLKDENTGYILYEDTNN 626
Vip3Ac1        GGVNGTKVLYVHEDGEFSQFIGDKLKLKTEYVIQYIVKGKAAIYLKDEKNGDYIYEETNN 623
Vip3Ad1        GGVNGTRALYVHKDGEFSHFIGDKLKSKTEYLIRYIVKGKASIFLKDEKNENYIYEDTNN 624
Vip3Ab1        GGINGTKVLYVHKDGEFSQFVGGKLKSKTEYVIQYIVKGKASIYLKDKKNENSIYEEINN 624
Vip3Aj1        GGVNGTRALYVHKDGEFSHFIGDKLKSKTEYLIRYIVKGKASIFLKDEKNENYIYEDTNN 624
Vip3Ae1        GGVKGTKVLYVHKDGEFSQFIGYKLKSKTEYVIQYIVKGKAVIYLKDEKNGDYIYEEINN 624
Vip3Ag1        GGVNGTKALYVHKDGEFSQFIGDKLKSKTEYVIQYIVKGKASILLKDEKNGDCIYEDTNN 624
               . :     *:.: ** . :*:* *** :*:*:*   :**   : **::      :*: ..

Vip3Bc1        NFSDFQTVTKKFNSGVNPSEIYFLFKNQSEYEAWGNNFIILEIKSLEF--LPQMLKPEDW 701
Vip3B2160      NFSDFQTVTKKFNSGVNPSEIYFLFKNQSEYEAWGNNFIILEIKSLEF--LPQMLKPEDW 692
Vip3Ba1        NFDTFQTITKKFNSGVDPSEIYLVFKNQIGYEAWGNNFIILEIKSLET--LPQILKPENW 702
Vip3Bb1        KFDTFQTITKKFNSGVDPSEIYLVFKNQIGYEAWGNKFIILEIKSFET--LPQILKPENW 700
Vip3Ca1        NSEEFQTIAVKFTSETDLSQTHLVFKSQNGYEAWGDNFIILEAKLFETPESPELIKFNDW 692
Vip3Ah1        NLEAFQTITKRFTTELDSSDVYLVFKCKNGYKAWGDNFLITEIRPKE-VVSPELIKVENW 683
Vip3Aa1        NLEDYQTINKRFTTGTDLKGVYLILKSQNGDEAWGDNFIILEISPSEKLLSPELINTNNW 684
Vip3Aa16       NLEDYQTINKRFTTGTDLKGVYLILKSQNGDEAWGDNFIILEISPSEKLLSPELINTNNW 684
Vip3Af1        NLKDYQTITKRFTTGTDLKGVYLILKSQNGDEAWGDKFTILEIKPAEDLLSPELINPNSW 684
Vip3Ai1        DLEDFQTITKRFTTGTDLMRVYLILKSQSGHEAWGDNFTILEIKPAEALVSPELINPNSW 686
Vip3Ac1        ELEDFQAVTKRFITGTDSSRVHLIFTSQNGEEAFGGNFIISEIRPSEELLSPELIKSDAW 683
Vip3Ad1        NLEDYQTITKRFTTGTDSTGVYLIFNSQNGDEAWGDNFIILEISPCEKLLSPELIKTDKW 684
Vip3Ab1        DLEGFQTVTKRFITGTDSSGIHLIFTSQNGEGAFGGNFIISEIRTSEELLSPELIMSDAW 684
Vip3Aj1        NLEDYQTITKRFTTGTDSTGVYLIFNSQNGDEAFGENFTISEIRLSEDLLSPELINSDAW 684
Vip3Ae1        ELEDFQTVTKRFITGTDSSGVHLIFTSQNGEEAFGGNFIISEIRPSEELLSPELIKSDAW 684
Vip3Ag1        GLEDFQTITKSFITGTDSSGVHLIFNSQNGDEAFGENFTISEIRLSEDLLSPELINSDAW 684
                 . :*::   * :  :    ::::. :    *:* :* * *    *    *:::  : *
```

```
                                                                      K754
                                                                         |
Vip3Bc1       IPSGNVQMKDGGR--LEILGDGYFKQFIKLENDSTYHLRLSV--KGTGRVSIIDESKYLL 757
Vip3B2160     IPSGNVQMKDGGR--LEILGDGYFKQFIKLENDSTYHLRLSV--KGTGRVSIIDESKYLL 748
Vip3Ba1       IPLGNAEIKEDGK--IEISGNGSMNQYIQLEQNSKYHLRFSV--KGKGRVTMQAQTSHI- 757
Vip3Bb1       MPFGNAEIKEDGK--IEISGNGTMTQNIQLEQNSKYHLRFSV--KGKGRVAIQTQSSHI- 755
Vip3Ca1       ERFGTTY-ITGNELRIDHSRGGYFRQSLNIDSYSTYDLSFSFS-GLWAKVIVKNSRGVVL 750
Vip3Ah1       IGMGGSNHVNPDSLLLFTGGRSILKQNLQLDSYSTYRVRFSLMVIGKAKVIIRNSSEVLF 743
Vip3Aa1       TSTGSTN-ISGNTLTLYQGGRGILKQNLQLDSFSTYRVYFSV--SGDANVRIRNSREVLF 741
Vip3Aa16      TSTGSTN-ISGNTLTLYQGGRGILKQNLQLDSFSTYRVYFSV--SGDANVRIRNSREVLF 752
Vip3Af1       ITTPGAS-ISGNKLFINLGTNGTFRQSLSLNSYSTYSISFTA--SGPFNVTVRNSREVLF 741
Vip3Ai1       ITTQGAS-ISGDKLFISLGTNGTFRQNLSLNSYSTYSISFTA--SGPFNVTVRNSREVLY 743
Vip3Ac1       VGSQGTW-ISGNSLNINSNVNGTFRQNLSLESYSTYSMNFNV--NGFGKVTIRNSREVVF 740
Vip3Ad1       ISTGSTY-ISDDRLTLYQGGRGILKQNLQLDRFSTYRVNFSV--NGDANVRIRNSREVLL 741
Vip3Ab1       VGSQGTW-ISGNSLTINSNVNGTFRQNLPLESYSTYSMNFTV--NGFGKVTVRNSREVLF 741
Vip3Aj1       VGSQGTW-ISGNSLTINSNVNGTFRQNLSLESYSTYSMNFNV--NGFAKVTVRNSREVLF 741
Vip3Ae1       VGTQGAW-NSGNSLTIYTNTNGTFRQNLPLESYSTYSMNFNI--TGFGKVTIRNSREVLF 741
Vip3Ag1       VGSQGTW-ISGNSLTINSNVNGTFRQNLSLESYSTYSMNFNV--NGFAKVTVRNSREVLF 741
                         .   :     . : * : ::  *.* : :.       .* :  .   : 

Vip3Bc1       FVNV------KDEDLTRVIKNTSSKGECFIALEGT--YVENSSTIFSNVSIVKE       803
Vip3B2160     FVNV------KDEDLTRVIKNTSSKGECFIALEGT--YVENSSTIFSNVSIVKE       794
Vip3Ba1       NVPA------TNEEVSIMIETTRLYGEGIISLLND--EVENSGVIFSDVSIVKE       803
Vip3Bb1       NVPA------TNEEVSTMITTRNLYGEGMIYLFND--DVENSKVIFSDVSLVKE       801
Vip3Ca1       FEKV-KNNGSSYEDISESFTTASNKDGFFIELTAERT--SSTFHSFRDISIKEKIE     803
Vip3Ah1       EKSYVNDSEGVLEGVSETFTTKSIQDNFYVELSNEGTFGSKDVAYFYNFSIK         795
Vip3Aa1       EKRY-MSGA---KDVSEMFTTKFEKDNFYIELSQGNNLYGGPIVHFYDVSIK         789
Vip3Aa16      EKRY-MSGA---KDVSEMFTTKFEKDNFYIELSQGNNLYGGPIVHFYDVSIK         789
Vip3Af1       ERSNLMSST---SHISGTFKTESNNTGLYVELSRRSG--GGGHISFENVSIK         788
Vip3Ai1       ERNNLMSST---SHISGEFKTESNNTGLYVELSRRSG--GAGHISFENISIK         790
Vip3Ac1       ERSYLQFSS---KYISEKFTTTTNNTGLYVELSRASS---RGVINFGDFSIK         786
Vip3Ad1       EKRYL--NR---KGVSEMFTTKFDKDNFYVELSQGDNL--GTVVHFYDFSIK         786
Vip3Ab1       EKSYPQLSP---KDISEKFTTAANNTGLYVELSRSTS---GGAINFRDFSIK         787
Vip3Aj1       EKNYPQLSP---KDIFEKFTTAANNTGLYVELSRFTS---GGAITFRDFSIK         787
Vip3Ae1       EKNFSQLSP---KDYSEKFTTAANNTGFYVELSRGTQ---GGNITFRDFSIK         787
Vip3Ag1       EKNYPQLSP---KDISEKFTTAANNTGLYVELSRFTS---GGAINFRNFSIK         787
                          .     : .        : *             * :.*:
```

**Supplementary Figure 4- Sequence alignment of Vip3 toxins.** Alignment generated by CLUSTAL O(1.2.4). Residues divergent from the Vip3Bc1 reference sequence are highlighted with a separate colour for each different residue. Below the sequence, similarity is shown according to Clustal parameters and coloured, by domain as in Figure 1, with domain 1 in cyan, domain 2 in blue, domain 3 in green, domain 4 in yellow, domain 5 in red. Holotype sequences are in black text and sequences associated with recent structural studies are coloured: Vip3B2160 (blue letters) is the sequence of the mutant Vip3B taken from the crystal structure 6V1V (numbered in accordance with this PDB entry); Vip3Aa16 (green letters) is the sequence for the structure with PDB code 6TFJ. Key features of the Vip3Bc1 sequence are highlighted including the primary trypsin cleavage site at K205, and minor trypsin processing site at K754.

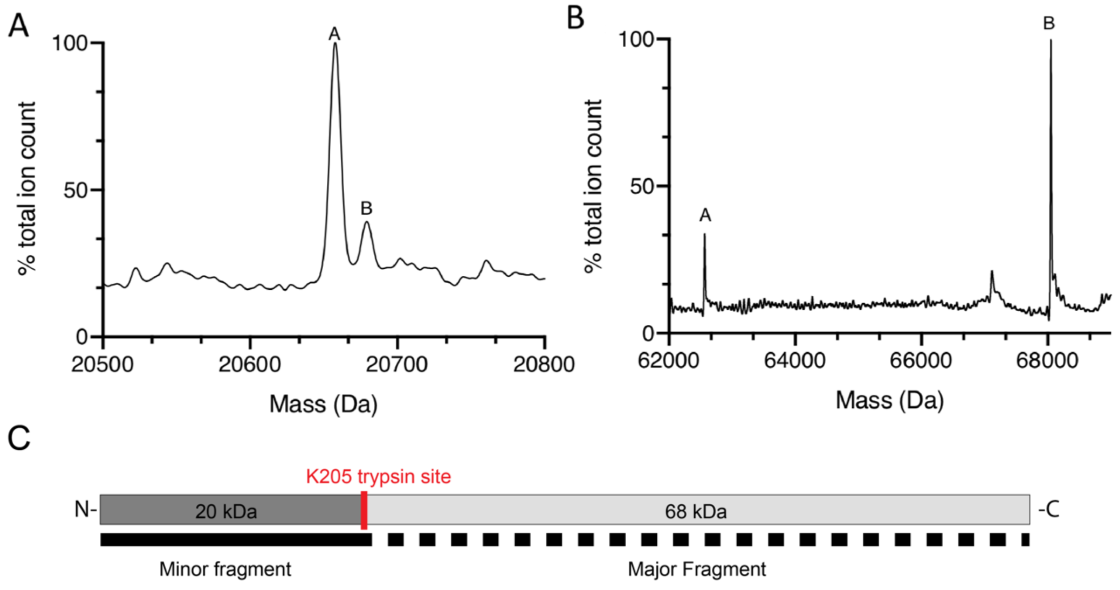


**Supplementary Figure 5- Mass spectra analysis of Vip3Bc1$^{act}$**. A) Mass spectrum analysis of ~ 20 kDa fragments, peak A corresponding to 20657.7 Da, B 20679 Da. B) Mass spectrum analysis of ~ 65 kDa fragments, peak A 62565.3, B 68050.7 Da. Trypsinisation at K205 results in formation of 20.65 kDa and 68.05 kDa species. Additional lower abundance species is seen at 62.56 kDa which corresponds to additional processing at K754. C) Schematic of Vip3 gene.

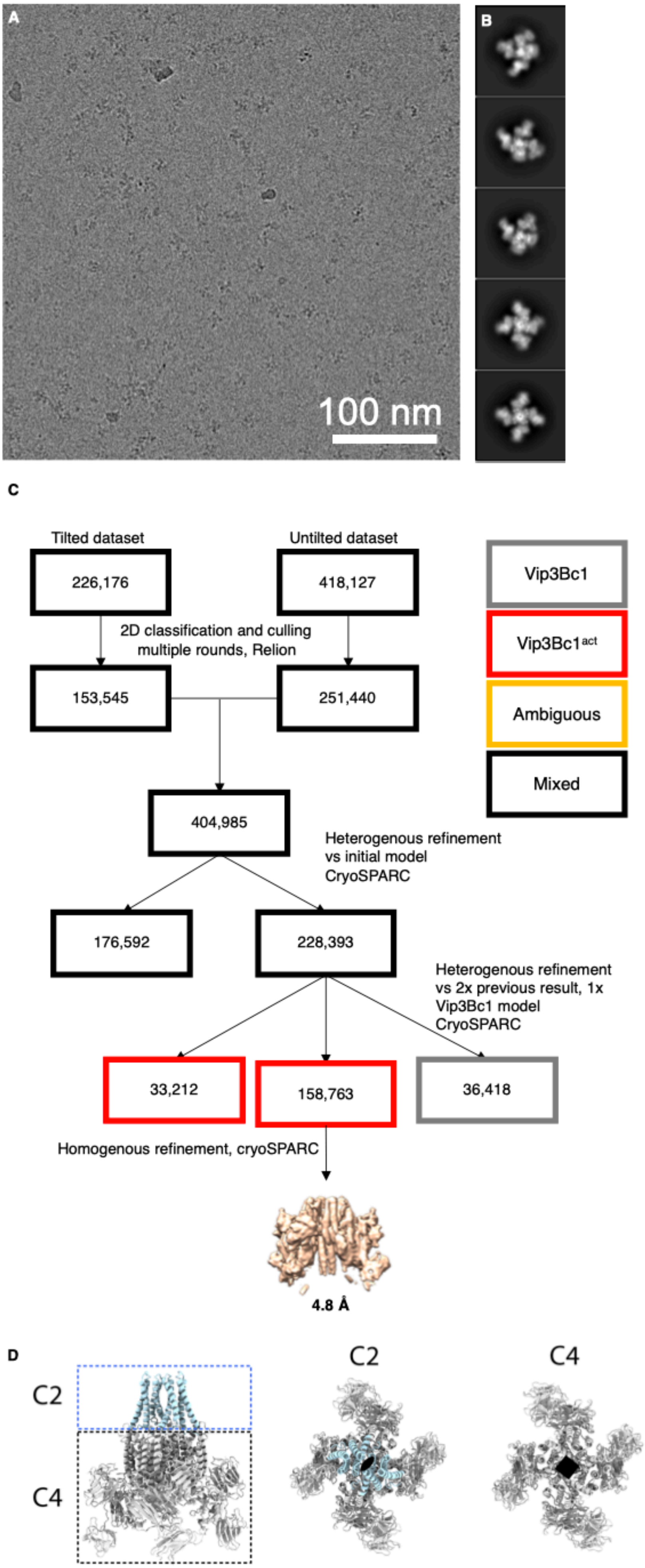
A
100 nm
B
C
Tilted dataset
226,176
Untilted dataset
418,127
2D classification and culling
multiple rounds, Relion
153,545
251,440
404,985
Heterogenous refinement
vs initial model
CryoSPARC
176,592
228,393
Heterogenous refinement
vs 2x previous result, 1x
Vip3Bc1 model
CryoSPARC
33,212
158,763
36,418
Homogenous refinement, cryoSPARC
4.8 Å
Vip3Bc1
Vip3Bc1act
Ambiguous
Mixed
D
C2
C4
C2
C4

**Supplementary Figure 6- Image processing of Vip3Bc1$^{act}$ dataset.** A) Representative micrograph from a dataset of 8051 images and B) representative 2D class averages showing a range of angular orientations.. C) Image processing pipelines for Vip3Bc1$^{act}$. Numbers indicate #particles at given stages during the reconstruction process. Reconstruction statistics can be seen in Supplementary table 1. D) Symmetry change from C2 to C4 during Vip3Bc1 to Vip3Bc1$^{act}$ structural rearrangements. Domain 1 (blue) undertakes two different conformations in Vip3Bc1 prior to activation while domains 2,3,4,5 undertake a single conformation in each of the 4 monomers. Following activation, domain one is no longer present in the resolved density, whilst domains 2,3,4,5 are – and retain C4 symmetry (right most panel). Ellipse denotes 2-fold symmetry axis. Square denotes 4-fold symmetry axis.

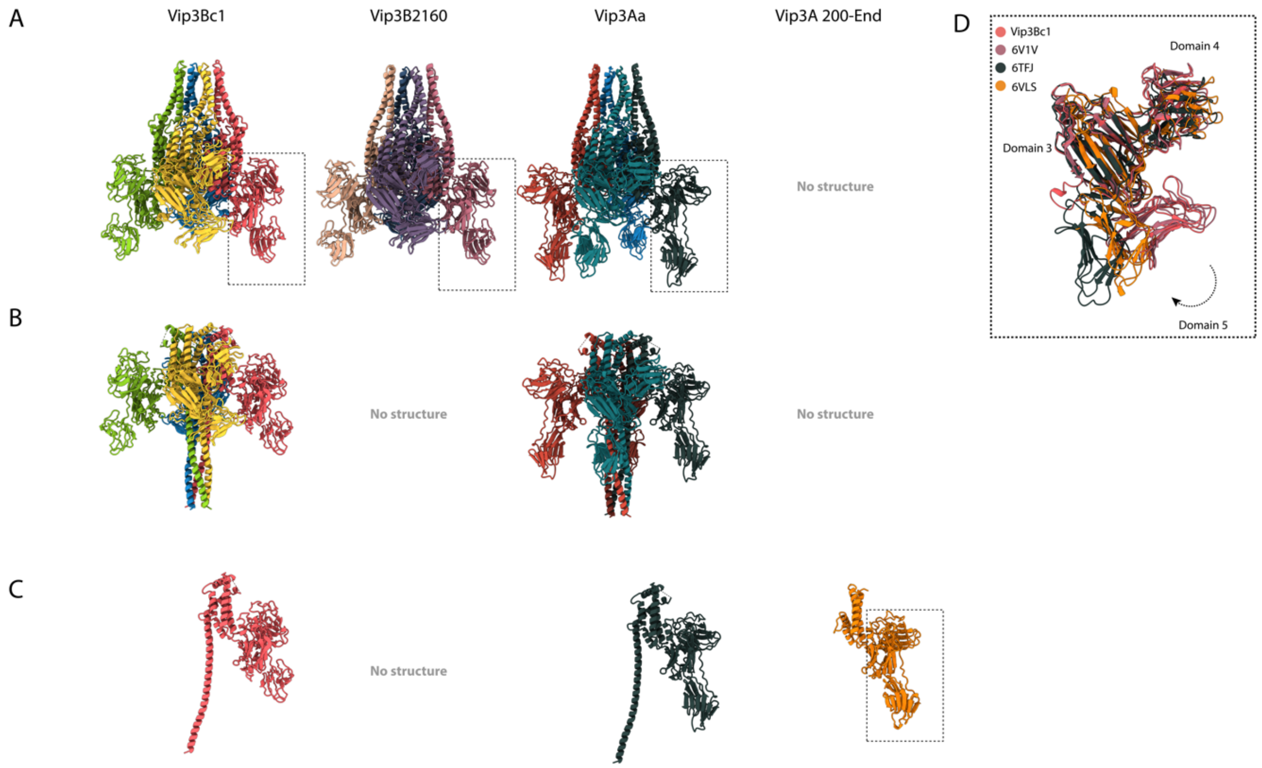


**Supplementary Figure 7. Comparison of Vip3 Structures to date.** A) Pro-toxin structures of Vip3Bc1 (this study), Vip3B2160 (6V1V) and Vip3Aa16 (6TFJ) exhibit similar structures with a tetrameric supramolecular assembly. B) Activated structures of Vip3Bc1 (this study) and Vip3Aa16 (PDB 6TFK). C) Monomers of activated Vip3Bc1 (this study) and Vip3Aa (PDB 6TFK) alongside the crystal structure of truncated Vip3a (PDB 6VLS) demonstrate the absence of the pore forming domain 1 in PDB 6VLS, indicating it is unlikely to be in a biologically relevant conformation. D) Structural superposition of domains 3-5 from each of the Vip3 structures shows that domain 5 takes varying conformations relative to domains 3 and 4 across Vip3 variants.

**Supplementary Movie 1- Cryo-ET of Vip3Bc1$^{act}$ complex binding to membrane in the 'umbrella' conformation.** Animation of tomogram shown in Figure 4 with the filtered tomogram revealing segmentation of the density.

**Supplementary Movie 2- Cryo-ET of Vip3Bc1$^{act}$ and LUV preparation, cropped around single particle to highlight membrane perturbation.**

**Supplementary Movie 3- Cryo-ET of Vip3Bc1$^{act}$ showing a large number of particles bound on a single LUV.**

**Supplementary Movie 4- Cryo-ET of Vip3Bc1 and LUV preparation, where individual Vip3Bc1 particles cannot be seen, but protein aggregates are observed.**

| | #1 Vip3Bc1 (EMD-10888) (PDB 6YRF) | #2 Vip3Bc1$^{act}$ (EMDB-10889 (PDB 6YRG) (Two independent collections) |
|---|---|---|
| **Data collection and processing** | | |
| Nominal magnification | 75,000 | 75,000 |
| Voltage (kV) | 300 | 300 |
| Electron exposure (e/Å) | 76.7 | 70.8 /42.51 |
| Defocus range (μm) | -1.3 to -3.0 | -1.5 to -3.0 |
| Pixel size (Å) | 1.065 | 1.065 |
| Exposure time (s) | 1.5 | 1.5 /1 |
| Number of frames | 59 | 59 |
| Number of images | 17900 | 5048/ 3003 |
| Tilt angle | 0° | 0° /10-20° |
| Symmetry imposed | C2 | C4 |
| Initial particle images (no.) | 1414999 | 664303 |
| Final particle images (no.) | 397437 | 158763 |
| Map resolution (Å) | 3.94 | 4.75 |
| FSC threshold | 0.143 | 0.143 |
| B-factor applied | -257.5 | -287.8 |
| **Refinement** | | |
| Initial model used (PDB code) | 6V1V | 6TFK, 6V1V |
| Model composition | | |
| Protein residues | 3108 | 2736 |
| Ligands | 0 | 0 |

| | | |
|---|---|---|
| R.m.s. deviations | | |
| Bond lengths (Å) | 0.008 | 0.005 |
| Bond angles (°) | 0.903 | 0.745 |
| Validation | | |
| MolProbity score | 2.55 | 2.51 |
| Clashscore | 27 | 30.52 |
| Ramachandran plot | | |
| Favored (%) | 87.1 | 90.33 |
| Allowed (%) | 12.77 | 9.19 |
| Disallowed (%) | 0.21 | 0.48 |

**Supplementary Table 1-** CryoEM parameters for single particle data collection sessions

| Figure | Figure 4/ Supplementary Movie 1 | Supplementary Movie 3 | Supplementary Movie 4 |
|---|---|---|---|
| **Sample** | **Vip3Bc1$^{act}$** | **Vip3Bc1$^{act}$** | **Vip3Bc1** |
| **Protein concentration (µg/mL)** | 60 | 60 | 60 |
| **Lipid concentration** | 750 µM | 450 µM | 750 µM |
| **Pixel size** | 2.7 | 3.42 | 2.7 |
| **Tilt angles** | +/- 60 | +/- 60 | +/- 60 |
| **Tilt increment** | 2 | 2 | 2 |
| **Dose scheme** | Dose symmetric | 0-60 > 0- -60 | Dose symmetric |
| **Single exposure time** | 2 | 3s | 2 |
| **Single exposure dose** | 1.26 | 1.8 e/Å2 | 1.26 |
| **Single exposure frames** | 3 | 3 | 3 |

**Supplementary Table 2-** CryoET parameters for tilt series collection

| Protein concentration (μM) | #1 | #2 | #3 |
|---|---|---|---|
| **0.006** | 2.57489 | 6.52635 | 2.68607 |
| **0.02** | 7.0462 | 1.03595 | 0.66029 |
| **0.06** | 5.01458 | 1.82574 | 0.39485 |
| **0.2** | 5.40889 | 2.85248 | 4.47018 |
| **0.6** | 3.55127 | 5.4101 | 4.65638 |
| **2** | 9.34069 | 8.9497 | 8.70266 |
| **6** | 6.86094 | 11.43888 | 6.96873 |
| **20** | 17.4509 | 18.91824 | 19.35714 |
| **60** | 27.38118 | 26.3002 | 24.95642 |
| **Triton control** | 100 | 100 | 100 |

**Supplementary Table 3-** Normalised (%)(Triton treated 100%) data from liposome dye release assays for each replicate for Vip3Bc1 treatment as shown in Figure 2A.

| **Protein concentration (μM)** | #1 | #2 | #3 |
|---|---|---|---|
| **0.006** | 5.27574 | 11.99272 | 10.78506 |
| **0.02** | 6.35520 | 9.87165 | 15.86453 |
| **0.06** | 14.94488 | 10.31583 | 19.67848 |
| **0.2** | 21.06937 | 31.61999 | 16.48555 |
| **0.6** | 35.31104 | 24.14328 | 37.45194 |
| **2** | 54.99839 | 40.00127 | 45.34057 |
| **6** | 51.16814 | 65.87692 | 53.13982 |
| **20** | 78.97468 | 57.32994 | 68.20661 |
| **60** | 63.58282 | 66.4246 | 73.72434 |

**Supplementary Table 4-** Normalised data (%)(Triton treated 100%) from liposome dye release assays for each replicate for Vip3Bc1$^{act}$ treatment as shown in Figure 2A.
